# A local cost simulation-based algorithm to solve distributed constraint optimization problems

**DOI:** 10.7717/peerj-cs.1296

**Published:** 2023-03-17

**Authors:** Meifeng Shi, Feipeng Liang, Yuan Chen, Ying He

**Affiliations:** 1 Department of Computer Science and Engineering, Chongqing University of Technology, Chongqing, China; 2 Faculty of Information Science and Electrical Engineering, Kyushu University, Fukuoka, Japan

**Keywords:** DCOPs, LCS, EWMA, Inferior solutions

## Abstract

As an important incomplete algorithm for solving Distributed Constraint Optimization Problems (DCOPs), local search algorithms exhibit the advantages of flexibility, high efficiency and high fault tolerance. However, the significant historical values of agents that affect the local cost and global cost are never taken into in existing incomplete algorithms. In this article, a novel Local Cost Simulation-based Algorithm named LCS is presented to exploit the potential of historical values of agents to further enhance the exploration ability of the local search algorithm. In LCS, the Exponential Weighted Moving Average (EWMA) is introduced to simulate the local cost to generate the selection probability of each value. Moreover, populations are constructed for each agent to increase the times of being selected inferior solutions by population optimization and information exchange between populations. We theoretically analyze the feasibility of EWMA and the availability of solution quality improvement. In addition, based on our extensive empirical evaluations, we experimentally demonstrate that LCS outperforms state-of-the-art DCOP incomplete algorithms.

## Introduction

Multi-agent system (MAS) (Ferber & Weiss, 1999) is a computing system composed of multiple autonomous agents that can interact with each other. It is an important research area of distributed artificial intelligence. The Distributed Constrained Optimization Problems (DCOPs) (Modi et al., 2005) have emerged as one of the main coordination techniques in MAS. It is based on the constrained relationship between agents to make decisions and select a set of values with minimum constraint cost. DCOPs have been successfully applied in real-world coordination tasks, such as sensor networks (Farinelli, Rogers & Jennings, 2014), task scheduling (Sultanik, Modi & Regli, 2007), power networks (Fioretto et al., 2017), *etc.*

Algorithms for DCOPs can be classified into complete algorithms that guarantee to find of the optimal solution and incomplete algorithms that can find the approximate optimal solution in a short time. The complete algorithms can be roughly classified into the search-based algorithm (Gershman, Meisels & Zivan, 2009) by traversing the solution space and inference-based algorithm (Petcu & Faltings, 2005; Petcu & Faltings, 2007) by performing variable elimination. Complete algorithms based on search mainly include SynchBB (Hirayama & Yokoo, 1997), AFB (Gershman, Meisels & Zivan, 2009) and ADOPT (Modi et al., 2005). Among them, SynchBB is a synchronous algorithm, which uses the idea of branch delimitation to solve DCOP, mainly uses the boundary value to prune the solution space and then compresses the space to find the optimal solution. AFB is an asynchronous algorithm, which is an improved version of SynchBB. In AFB, the agent assigns values to variables sequentially and transmits the partial resolution and the cost of the partial solutions. By receiving the information, the agent asynchronously calculates the upper and lower bounds of the current partial solution, so that it can judge whether backtracking is needed as early as possible, which improves the pruning efficiency of the algorithm. ADOPT adopts a depth-first search tree as a communication structure, and uses a best-first search strategy to perform distributed backtracking. The main representative of the complete algorithm based on inference is DPOP (Petcu & Faltings, 2005), which uses pseudo-tree (Freuder & Quinn, 1985) as the communication structure, calculates and transmits the utility corresponding to the assignment combination from the bottom to top, and obtains the optimal solution through the elimination operation of the joint utility tables. Since DCOPs are NP-Hard (Modi et al., 2005), the computational overheads of complete algorithms will increase exponentially as the scale of the problem increases, which limits their application in practical engineering.The advantage of low computational overheads of incomplete algorithms makes them more popular in large-scale applications.

Incomplete algorithms are generally classified into three categories: local search algorithms, inference-based algorithms, and sampling-based algorithms. Inference-based algorithms mainly include Max-Sum (Farinelli et al., 2008), Bounded Max-sum (Rogers et al., 2011), Max-sum_ADVP (Zivan et al., 2017) and Damped Max-sum (Cohen & Zivan, 2017). In Max-sum, the agent optimizes the goal by passing messages in the factor graph (Aji & McEliece, 2000) to continuously accumulate beliefs. Bounded Max-sum transforms the original problem into an acyclic graph by removing the relationship edges that have the least impact on the solution quality in the cyclic factor graph. The problem is then solved using the Max-sum algorithm and an approximation ratio is calculated to improve the quality of the solution. Max-sum_ADVP solves the repeated utility problem by adding its own fetch values to the information sent by each variable node and eliminates the assumption of invalid value. Damped Max-sum converts the standard factor graph into an equivalent split-constrained factor graph to control the asymmetry of each constraint, in order to achieve the purpose of balancing exploration and utilization.

Sampling-based algorithms include DUCT (Ottens, Dimitrakakis & Faltings, 2012), D-Gibbs (Nguyen, Yeoh & Lau, 2013), SD-Gibbs (Nguyen et al., 2019) and PD-Gibbs (Nguyen et al., 2019) that take statistical samples of the value context to solve DCOPs by constructing another structure. Among them, DUCT applies sampling and confidence bounds to solve DCOP. The main idea of D-Gibbs is to transform DCOP into a maximum likelihood estimation problem in Markov random fields. SD-Gibbs and PD-Gibbs have a linear-space memory requirement and solve some large memory limitation problems.

The local search algorithms are the most popular ones because of their simple logic, flexibility and effectiveness. Local search algorithms mainly include DSA (Zhang et al., 2005), MGM (Maheswaran, Pearce & Tambe, 2004), DSAN (Arshad & Silaghi, 2004), MGM-2 (Maheswaran, Pearce & Tambe, 2006), MGM-3 (Maheswaran, Pearce & Tambe, 2006; Leite, Fabrício & Jean-Paul, 2014) and GDBA (Okamoto, Zivan & Nahon, 2016). DSA records the current local cost according to the values of neighbors and selects the value that can reduce the local cost to the greatest extent according to the probability. MGM considers the local cost of neighbors, and the agent with the largest local gain can change the value. DSAN introduces the dynamic probability idea of the simulated annealing method to jump out of the local optimum. Algorithms such as MGM-2 and MGM-3 use the idea of k-optimal (Bowring et al., 2008), agents cooperate locally to improve the quality of local solutions, but the larger k is, the greater the time complexity will be. GDBA extends DBA (Zhang et al., 2005) to DCOP, stipulates concepts such as effective cost, constraint violation and modified matrix range, and has achieved good results. In the iterative process of the local search algorithm, although the total cost of the solution shows a downward trend, it does not decrease monotonically. After falling to the lowest value, since the agent does not know the current global cost state, the agent will continue to change the value according to the local strategy. Therefore, in order to record the optimal solution in the iterative process and make the algorithm show anytime effect, the Anytime Local Search Framework (Zivan, Okamoto & Peled, 2014) was proposed. The framework relies on a breadth-first tree structure to record the global state. In addition, PDS (Yu et al., 2017) also proposes a partial decision-making framework. It is able to apply local search algorithms and improve the quality of local search algorithm solutions by local decision mechanisms.

Recently, population-based methods and frameworks have emerged and can achieve high-quality solutions. ACO_DCOP (Chen et al., 2018) adapts the traditional Ant Colony Optimization to solve DCOPs by the pheromone mechanism ameliorated. ACO_DCOP defines pheromone as global cost and heuristic information as local cost, and the agent combines the information from both parts to make a decision. A genetic algorithm-based (LSGA) (Chen et al., 2020) framework uses genetic codes to crossover variation the assignment combination.

However, as the iterative trace of the algorithm, the historical values of agents are never considered in the previously mentioned algorithms. To study the influence of the historical values of agents on local search algorithms, a novel Local Cost Simulation-based algorithm named LCS is proposed by combining the Exponentially Weighted Moving Average (EWMA) and population optimization technology, which improves the development of solutions and exploration capabilities. First, EWMA is used to simulate the local cost to generate the selection probability of each value. Then, populations are constructed for each agent to increase the selection times of inferior solutions. Finally, to break out of the local optima to explore other solution spaces, the population interaction is introduced into LCS. We theoretically analyze the feasibility of EWMA, availability of solution quality improvement of the proposed LCS. We also experimentally show the superior performance of LCS over the state-of-the-art DCOPs incomplete algorithms based on our extensive empirical evaluations. Specically, our contributions can be summarized as follows:

(a) We use EWMA to model historical local costs, which provides an effective idea for using historical values to estimate the local cost.
(b) We design a probability-based strategy so that values with lower local cost simulation values have a higher probability of being selected.
(c) We design a population cooperation mechanism to improve the exploitation and exploration ability of LCS.

## Background & Related work

### Distributed constraint optimization problems

A DCOP can be defined as a tuple (A, X, D, F) (Chen et al., 2020) where.

• A = {a_1_, …, a_n_} is a set of agents.
• X = {x_1_, …, x_m_} is a set of variables, where each variable is assigned to an agent.
• D = {D_1_, …, D_m_} is a set of finite variable domains, where the values of the variables are taken from finite domains D_1_, …, D_m_.
• F = {f_1_, …, f_q_} is a set of constraints, where each constraint f_i_:D_i_1__ × … × D_i_k__ → ℝ_≥0_ specifies a non-negative cost for every possible value combination of a set of variables.

**Figure 1:**
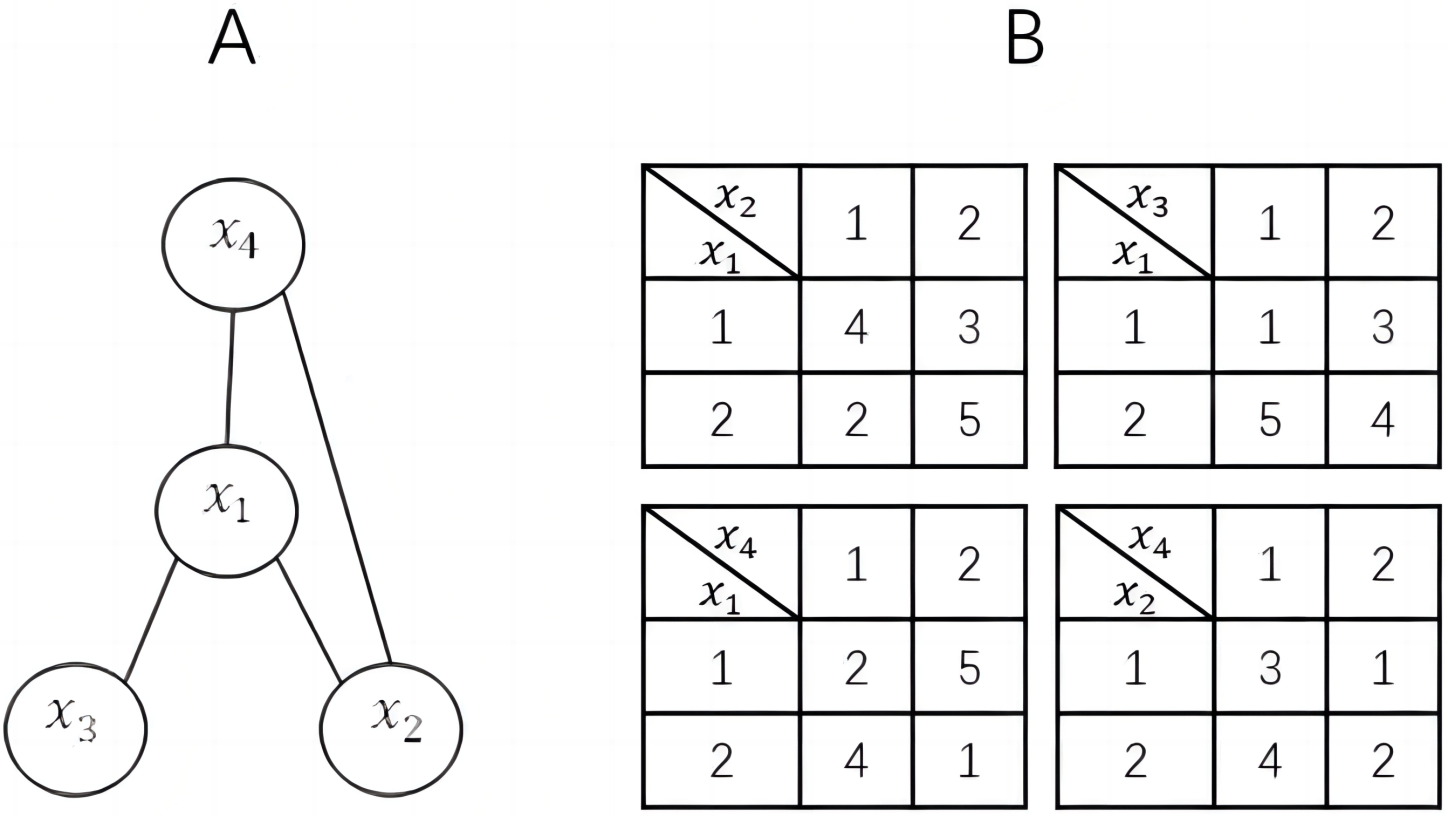
A DCOP instance. (A) constraint graph; (B) constraint matrices.

For ease of understanding and discussion, we assume that one agent controls only one variable and all constraints are binary. Therefore, in this article, agent and variable can be considered as the same concept and can be replaced with each other. Figure 1 shows a simple DCOP example.

The solution objective of DCOP is to find an assignment X^∗^ that minimizes the sum of constraint costs arising between all agents.

(1) \begin{eqnarray*}{\mathrm{X}}^{\ast }=\text{argmin}_{{\mathrm{d}}_{\mathrm{ i}}\in {\mathrm{D}}_{\mathrm{i}},{\mathrm{d}}_{\mathrm{j}}\in {\mathrm{D}}_{\mathrm{j}}}\sum _{{\mathrm{f}}_{\mathrm{ij}}\in \mathrm{F}}{\mathrm{f}}_{\mathrm{ij}}({\mathrm{x}}_{\mathrm{i}}={\mathrm{d}}_{\mathrm{i}},{\mathrm{x}}_{\mathrm{j}}={\mathrm{d}}_{\mathrm{j}}).\end{eqnarray*}

### Local search framework for DCOPs

The local search algorithms are typical incomplete algorithms for solving DCOP problems. In each round, an agent sends the message that includes value or other information to neighbors in the constraint graph and receives the messages from neighbors. Then, the agent will select a new value in terms of the messages and decide whether to replace the old one according to different replacement strategies. There are different ways to create messages in different algorithms. In DSA, when the value of neighbors changes, the agent searches its domain to find a value that can reduce local cost, and replaces the old value using the new one with a probability. But in MGM, only the agent with maximal gain among all its neighbors can replace old value. In this article, DSA is used to illustrate this framework.

Table 1 presents the sketch of DSA. Firstly, the agent initializes a value randomly and sends it to all neighbors (line 1–2). Then, the algorithm executes a repeated iterative loop (line 3–9) until the termination condition is met. During the loop, an agent collects the values received from neighbors and selects a new value that reduces the local cost most (line 4–5). Finally, the agent decides to assign the new or the old value according to a probability p (line 7–9). If (Δ ≥ 0andp ≤ random()), assign the new value. Else, assign the old value.

### Related work

Most of incomplete algorithms for DCOPs are context-free (Deng et al., 2021). The local search algorithms (Chapman et al., 2011) is defined as: at each iteration, agents communicate only the states of the variables under their control to their neighbours on the constraint graph, and that reason about their next state based on the messages received from their neighbours.

In the most typical local search algorithm DSA, an agent sends its value to its neighbors, and at the same time receives the value sent from neighbors. Then, according to the received value, each agent chooses a value from its variable domain that can minimize the local cost to replace the old one with probability. If the the old value is replaced, the agent will resend it to its neighbors. In another typical algorithm, MGM, all agents need to send additional gain messages to their neighbors. Then through the message passing mechanism, only the agent with the largest gain among all neighbors can replace the old value. GDBA extends the DBA algorithm to the general DCOP problem and spans 24 combinations of three design choices. The three designs are strategies to modify the basic cost using multiple weights or additional penalties, definitions of constraint violations such as non-zero cost, non-minimal cost and maximum cost, and modifying the scope of the cost table like entries, rows, columns, or tables during breakthrough. DSAN is an improved version of DSA by applying simulated annealing to make DSA jump out of the local optimum.

The LCS proposed in this article mainly utilizes the historical information that current local search algorithms ignore. The exponential weighted moving average method is used to maintain and update the local cost simulation value, and the population cooperation mechanism is used to fully search the local cost simulation value, thereby improving the quality of the solution.

**Table 1:** The sketch of DSA.

**Algorithm 1** Distributed Stochastic Algorithm
**For each agent** ***x***_*i*_ **executes:**
1. value ←*Choose*_*Random*_*Value*()
2. send value to neighbors
3. **while** (no termination condition is met)
4. collect neighbors’ value
5. select a new value which reduces the local cost most
6. Δ← the number of the local cost reduced by the new value
7. **if**(Δ ≥ 0*andrandom*() ≤ *p*)**then**
8. assign the new value
9. send value to neighbors

## Proposed Method

### Motivation

In local search algorithms, only two successive rounds of information are taken into account and the historical information from previous rounds is wasted. For the DSA, the current value depends on the local cost difference between the current round and the previous round. That is, the value only depends on changes in a single round of neighbors’ values. The local cost is non-increasing in this strategy, and it is easy to fall into a local optimum. MGM follows the main idea of DSA, although the interactive competition between neighbors has been added to determine the value changes. For DSAN (Arshad & Silaghi, 2004), only the idea of Simulate Anneal Arithmetic (SAA) is introduced to control the search process to disturb the assignment probability when the algorithm falls into the local optima. The same is true for PDS, LSGA, *etc.* All of them make no use of the series of changes in local cost and its historical records of values.

In fact, for the local search algorithms, the purpose of the agent to change values is generally to reduce the local cost. At the time of local cost decreases, global cost always performs a downward trend as rounds increase. Various improved local search algorithms all focus on the conflict between local cost and global cost because when the global cost decreases while the local costs of some agents increase.

These improvement strategies are committed to making algorithms break away from the monotonic decline of local cost to jump out of the local optima and search for higher-quality solutions. All of these improved algorithms have not taken into account the change records of the local cost. The sum of the local costs of all agents is equal to the global cost of the system. It can be seen that the change of global cost is closely related to that change of the local cost. Suppose that the estimated local cost of each value is known, we can select the lower one to get a lower global cost. There are different local costs and different changes for different values. Thus, it is necessary to estimate the local cost for each value in the domain according to the historical value of agents to guide their assignments. Based on this, we propose a novel algorithm named LCS to solve DCOP based on the idea of local cost simulation.

### LCS

LCS consists of two phases: initialization and local cost simulation. We modify the existing ALS framework to make it compatible with the population of LCS. The sketch of LCS can be found in Table 2. Each value in the agent value range has a local cost simulation value that needs to be maintained, and the simulation value is the simulation value of the historical local cost generated when the value in the value range is selected. When the value of a_i_ is d_i_, the local cost simulation value of d_i_ is denoted as $\mathrm{est} \left( {\mathrm{d}}_{\mathrm{i}} \right) $.

**Table 2:** The sketch of LCS.

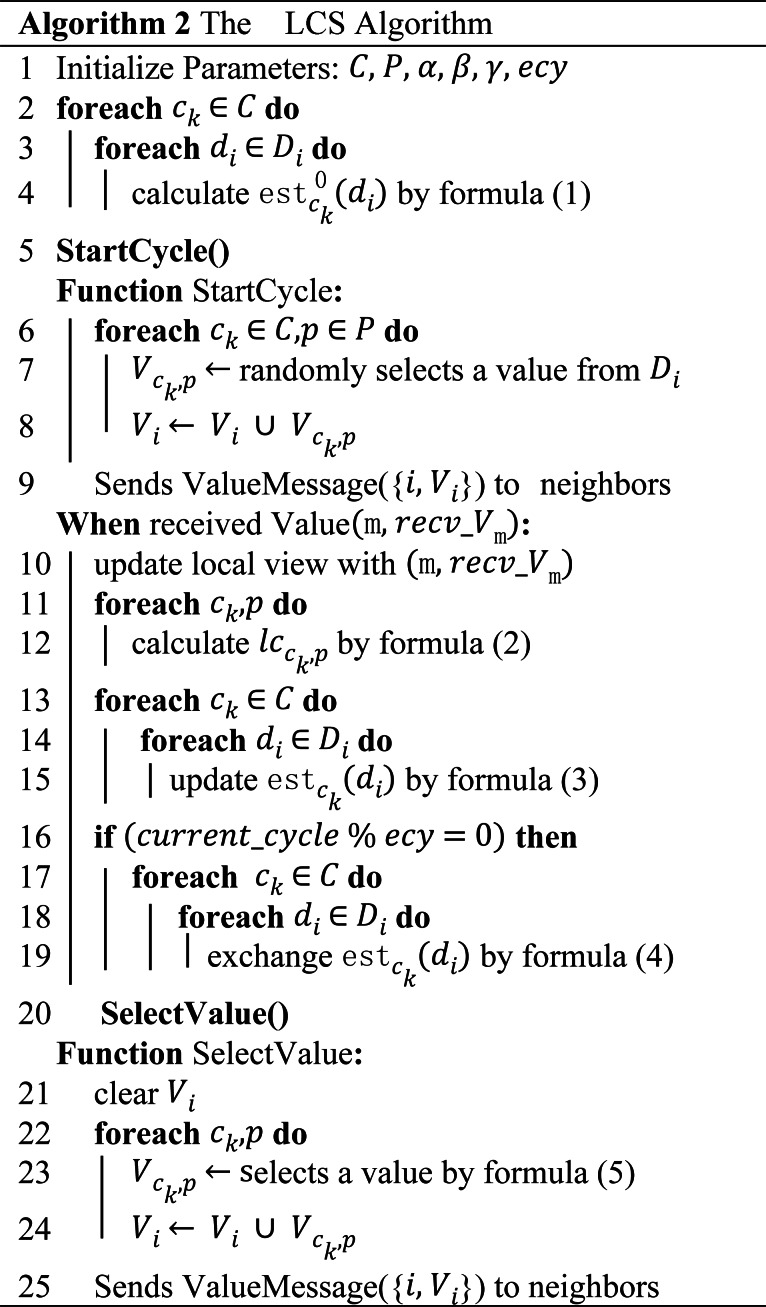

**Definition 1:** An individual is a value with a serial number. Individuals with the same serial number in all agents represent a solution. A population is a collection of individuals with its own unique local cost simulation.

**Definition 2:** $\mathrm{es}{\mathrm{t}}_{{\mathrm{c}}_{\mathrm{k}}}{ \left( {\mathrm{d}}_{\mathrm{i}} \right) }^{\mathrm{r}}$ is the simulation (or estimation) of the local cost that d_i_ ∈ D_i_ will produce. Where c_k_ ∈ C is the serial number of the population, and r is the number of simulation updates.

Initialization initiates the simulation and assigns values to individuals. Suppose that there are C populations, each of which has P individuals (C and P are the hyper-parameters). Since the algorithm has not yet started, we create the same initial simulation for all populations. For agent a_i_, the initial simulations (estimations) can be calculated by:

(2) \begin{eqnarray*}\mathrm{es}{\mathrm{t}}_{{\mathrm{c}}_{\mathrm{k}}}^{0} \left( {\mathrm{d}}_{\mathrm{ i}} \right) =\sum _{\mathrm{m}\in {\mathrm{N}}_{\mathrm{i}}}\max _{{\mathrm{d}}_{\mathrm{j}}\in {\mathrm{D}}_{\mathrm{m}}}\text{cost}({\mathrm{d}}_{\mathrm{i}},{\mathrm{d}}_{\mathrm{j}})\end{eqnarray*}

where ${\mathrm{est}}_{{\mathrm{c}}_{\mathrm{k}}}^{0} \left( {\mathrm{d}}_{\mathrm{i}} \right) $ is the initial estimation for c_k_ ∈ C. The estimation can simulate previous local costs and be updated according to the change of the local cost. It reflects the quality of d_i_ and records the change of neighbors’ values. Here it represents the worst case for d_i_. And N_i_ is the neighbor of a_i_, cost(d_i_, d_j_) isthe constraint cost generated when a_i_ takes the value of d_i_, and its neighbor a_j_ takes the value of d_j_.

Taking Fig. 1 for an example, we assume that there are two populations, with two individuals in each population respectively. The estimations for x_1_ should be calculated by:

\begin{eqnarray*}{\mathrm{est}}_{{\mathrm{c}}_{1}}^{0} \left( 1 \right) & ={\mathrm{est}}_{{\mathrm{ c}}_{2}}^{0} \left( 1 \right) =4+3+5=12 \end{eqnarray*}

\begin{eqnarray*}{\mathrm{est}}_{{\mathrm{c}}_{1}}^{0} \left( 2 \right) & ={\mathrm{est}}_{{\mathrm{ c}}_{2}}^{0} \left( 2 \right) =5+5+4=14 \end{eqnarray*}

After the estimations are initialized, each agent executes the StartCycle to start message-passing. The agent constructs populations and individuals to save values per round. We use V _i_ to represent the value set of populations for a_i_. At the first round, individual p ∈ P of population c_k_ ∈ C selects an initial value V _c_k_,p_ in the domain for itself randomly. After the selection of all individuals, the agent sends the set V _i_ with values to all neighbors.

For example in Table 3, after initialization, the individuals in each agent are randomly assigned.

Local cost simulation is a procedure to search for solutions. In this phase, the agent communicates continuously with its neighbors, calculates the local cost to update the simulated value, conducts population interaction to better simulate the local cost, and conducts a sufficient search for the potential of the value.

After the agent receives the sets V _m_ of neighbors, LCS needs to update simulations by local cost. The local cost for each individual can be calculated by:

(3) \begin{eqnarray*}{\mathrm{loc}}_{{\mathrm{c}}_{\mathrm{k}},\mathrm{p}}=\sum _{\mathrm{m}\in {\mathrm{N}}_{\mathrm{i}}}{\text{cost}}_{\mathrm{i},\mathrm{j}} \left( {\mathrm{V }}_{{\mathrm{c}}_{\mathrm{k}},\mathrm{p},\mathrm{i}},{\mathrm{V }}_{{\mathrm{c}}_{\mathrm{k}},\mathrm{p},\mathrm{m}} \right) \end{eqnarray*}

where loc_c_k_,p_ is the local cost which is calculated by individual p of population c_k_ and the same individuals of neighbors.

Continuing the example above, local costs for x_1_ can be calculated using:

\begin{eqnarray*}{\mathrm{loc}}_{{\mathrm{c}}_{1},{\mathrm{p}}_{1}}=3+3+2=8,{\mathrm{loc}}_{{\mathrm{c}}_{1},{\mathrm{p}}_{2}}=4+3+5=12& \end{eqnarray*}

\begin{eqnarray*}{\mathrm{loc}}_{{\mathrm{c}}_{2},{\mathrm{p}}_{1}}=3+1+5=9,{\mathrm{loc}}_{{\mathrm{c}}_{2},{\mathrm{p}}_{2}}=2+4+4=10.& \end{eqnarray*}

When the local cost of each individual has been obtained, the agent needs to update the estimation to simulate its change. Considering the temporal relationship between local cost and round, we use the EWMA to simulate the local cost. The updating method of estimation is defined as:

(4) \begin{eqnarray*}{\mathrm{est}}_{{\mathrm{c}}_{\mathrm{k}}}^{\mathrm{r}} \left( {\mathrm{d}}_{\mathrm{ i}} \right) ={\mathrm{est}}_{{\mathrm{c}}_{\mathrm{k}}}^{\mathrm{r}-1} \left( {\mathrm{d}}_{\mathrm{ i}} \right) \ast {\beta }_{{\mathrm{c}}_{\mathrm{k}}}+{\mathrm{loc}}_{{\mathrm{c}}_{\mathrm{k}},\mathrm{p}}\ast \left( 1-{\beta }_{{\mathrm{c}}_{\mathrm{k}}} \right) \end{eqnarray*}

where *β*_c_k__ is the decay rate of population c_k_, and r is the number of updates.

**Table 3:** Initialization example for population.

	V _c_1_,p_1__	V _c_1_,p_2__	V _c_2_,p_1__	V _c_2_,p_2__
V _1_	1	1	1	2
V _2_	2	1	2	1
V _3_	2	2	1	2
V _4_	1	2	2	1

Here, we assume that ${\beta }_{{\mathrm{c}}_{1}}=0.5,{\beta }_{{\mathrm{c}}_{2}}=0.6.{\mathrm{est}}_{{\mathrm{c}}_{\mathrm{k}}}^{0}$can be found at formulation 1. The estimations for x_1_ can be calculated as:

\begin{eqnarray*}{\mathrm{est}}_{{\mathrm{c}}_{1}}^{1} \left( 1 \right) & =12\ast 0.5+8\ast 0.5=10 \end{eqnarray*}

\begin{eqnarray*}{\mathrm{est}}_{{\mathrm{c}}_{1}}^{2} \left( 1 \right) & =10\ast 0.5+12\ast 0.5=11 \end{eqnarray*}

\begin{eqnarray*}{\mathrm{est}}_{{\mathrm{c}}_{2}}^{1} \left( 1 \right) & =12\ast 0.6+9\ast 0.4=10.8 \end{eqnarray*}

\begin{eqnarray*}{\mathrm{est}}_{{\mathrm{c}}_{2}}^{1} \left( 2 \right) & =14\ast 0.6+10\ast 0.4=12.4 \end{eqnarray*}

After the update, the simulations of x_1_ become: ${\mathrm{est}}_{{\mathrm{c}}_{1}} \left( 1 \right) ={\mathrm{est}}_{{\mathrm{c}}_{1}}^{2} \left( 1 \right) =11,{\mathrm{est}}_{{\mathrm{ c}}_{1}} \left( 2 \right) ={\mathrm{est}}_{{\mathrm{c}}_{1}}^{0} \left( 2 \right) =14,{\mathrm{est}}_{{\mathrm{ c}}_{2}} \left( 1 \right) ={\mathrm{est}}_{{\mathrm{c}}_{2}}^{1} \left( 1 \right) =10.8,{\mathrm{est}}_{{\mathrm{ c}}_{2}} \left( 2 \right) ={\mathrm{est}}_{{\mathrm{c}}_{2}}^{1} \left( 2 \right) =12.4$. If there is no superscript on est_c_k__(d_i_), it means est_c_k__(d_i_) is the current latest value.

After each population has updated its simulated values, a new estimate of the local cost that is closer to the actual local cost is obtained. At this time, in order to give full play to the advantages of the population, LCS sets up an exchange round, allowing the populations to share the simulated values they have found, which can tell those populations with poor simulated values that they have better values and better directions. to explore. The LCS pre-sets the exchange interval rounds, and every ecy rounds, the agent will exchange the obtained analog values. The purpose of the exchange operation is to share different search directions between populations. The exchange is defined as:

(5) \begin{eqnarray*}{\mathrm{est}}_{{\mathrm{c}}_{\mathrm{k}}} \left( {\mathrm{d}}_{\mathrm{i}} \right) ={\mathrm{est}}_{{\mathrm{c}}_{\mathrm{k}}} \left( {\mathrm{d}}_{\mathrm{i}} \right) \ast \left( 1-\gamma \right) +\min _{\mathrm{c} \in \mathrm{C}}~{\mathrm{est}}_{\mathrm{c}} \left( {\mathrm{d}}_{\mathrm{i}} \right) \ast \gamma \end{eqnarray*}

where *γ* is the learning rate. It can affect the reduction of estimation, by which the population can evolve to the best one.

Since just update, ${\min }_{\mathrm{c} \in  \mathrm{C}}~{\mathrm{est}}_{\mathrm{c}} \left( 1 \right) ={\mathrm{est}}_{{\mathrm{c}}_{2}}^{1} \left( 1 \right) =10.8,~{\min }_{\mathrm{c} \in  \mathrm{C}}~{\mathrm{est}}_{\mathrm{c}} \left( 2 \right) ={\mathrm{est}}_{{\mathrm{c}}_{2}}^{1} \left( 2 \right) =12.4$. We assume if *γ* = 0.5, the exchange values for x_1_ can be calculated by:

\begin{eqnarray*}{\mathrm{est}}_{{\mathrm{c}}_{1}} \left( 1 \right) & =11\ast 0.5+10.8\ast 0.5=10.9 \end{eqnarray*}

\begin{eqnarray*}{\mathrm{est}}_{{\mathrm{c}}_{1}} \left( 2 \right) & =14\ast 0.5+12.4\ast 0.5=13.2 \end{eqnarray*}

\begin{eqnarray*}{\mathrm{est}}_{{\mathrm{c}}_{2}} \left( 1 \right) & =10.8\ast 0.5+10.8\ast 0.5=10.8 \end{eqnarray*}

\begin{eqnarray*}{\mathrm{est}}_{{\mathrm{c}}_{2}} \left( 2 \right) & =12.4\ast 0.5+12.4\ast 0.5=12.4 \end{eqnarray*}

After being updated and exchanged, each individual executes a new round to select values. The agent needs to calculate the probability for each population since the estimations in populations are different. Reciprocal of estimation is used to get the probability. The probability for selecting value d_i_ is defined as:

(6) \begin{eqnarray*}{\mathrm{prb}}_{{\mathrm{c}}_{\mathrm{k}}} \left( {\mathrm{d}}_{\mathrm{i}} \right) = \frac{{ \left( 1/{\mathrm{est}}_{{\mathrm{c}}_{\mathrm{k}}}({\mathrm{d}}_{\mathrm{i}}) \right) }^{\alpha }}{\sum _{{\mathrm{d}}_{\mathrm{i}}^{{}^{{^{\prime}}}}\in {\mathrm{D}}_{\mathrm{i}}}{ \left( 1/{\mathrm{est}}_{{\mathrm{c}}_{\mathrm{k}}}({\mathrm{d}}_{\mathrm{i}}^{{}^{{^{\prime}}}}) \right) }^{\alpha }} \end{eqnarray*}

where *α* is a parameter that significantly affects the quality of the solution by enhancing the selected probability of d_i_. Because the range of estimation for different problems is distinct, it is necessary to set different *α*. Assume *α* = 8 here, the probability for c_1_ of x_1_ can be calculated by:

\begin{eqnarray*}{\mathrm{prb}}_{{\mathrm{c}}_{1}} \left( 1 \right) & = \frac{{ \left( 1/10.9 \right) }^{8}}{{ \left( 1/10.9 \right) }^{8}+{ \left( 1/13.2 \right) }^{8}} =0.822 \end{eqnarray*}

\begin{eqnarray*}{\mathrm{prb}}_{{\mathrm{c}}_{1}} \left( 2 \right) & = \frac{{ \left( 1/13.2 \right) }^{8}}{{ \left( 1/10.9 \right) }^{8}+{ \left( 1/13.2 \right) }^{8}} =0.178. \end{eqnarray*}

After finishing the new V _c_k_,p_ selection, the agent sends the new V _i_to neighbors again. At this time, one round is completed and the agent repeats the local cost simulation until all rounds are over. Because of the ALS, the optimal solution is x_1_ = 1, x_2_ = 2, x_3_ = 2, x_4_ = 1, which gets the lowest cost 8 in this round.

## Theoretical Analysis

### Theory

In this section, we prove the feasibility of EWMA, the availability of solution quality improvement, and the superiority of population interaction.

**Lemma 1.** EWMA will approach the actual local cost as the rounds go infinity.

**Proof.** Exponential weighted moving average means that the weighted coefficient of value decreases exponentially as time and can simulate the average value of a time series. The closer value is to the current moment, the greater the weighted coefficient is. In LCS, with the increasing of rounds the influence of previous local costs decreases, the average value obtained by EWMA can put less attention on it and focuses on the local costs in recent rounds. EWMA can alter the decay rate *β* to emphasize different rounds, the expression is:

(7) \begin{eqnarray*}{\mathrm{v}}_{\mathrm{t}}=\beta \ast {\mathrm{v}}_{\mathrm{t}-1}+ \left( 1-\beta \right) \ast {\theta }_{\mathrm{t}}\end{eqnarray*}

when v_0_ = 0, there are:

(8) \begin{eqnarray*}{\mathrm{v}}_{\mathrm{t}}= \left( 1-\beta \right) \left( {\theta }_{\mathrm{t}}+\beta {\theta }_{\mathrm{t}-1}+{\beta }^{2}{\theta }_{\mathrm{ t}-2}+\cdots +{\beta }^{\mathrm{t}-1}{\theta }_{1} \right) .\end{eqnarray*}

From formula (8), *θ* corresponds to the local cost, the weighting coefficient corresponding to *θ*_1_ is *β*^*t*−1^. When t is large, the coefficient value is close to 0 and decreases exponentially. The weighting coefficient corresponding to *θ*_t_ is 1, and the weight ratio is relatively large. There are multiple populations in the LCS, and different *β* can be set for the population. Both focus on different stages and control the diversity of searches.

**Proposition 1.** The quality of solutions improves as the number of message rounds increases before convergence.

**Proof.**What is the idea of LCS? The relationship between global cost and local cost is:

(9) \begin{eqnarray*}\mathrm{glc}= \frac{1}{2} \sum _{{\mathrm{a}}_{\mathrm{ i}}\in \text{Agent}}\mathrm{lo}{\mathrm{c}}_{{\mathrm{a}}_{\mathrm{i}}}\end{eqnarray*}

where glc is the global cost, loc_a_i__ is the local cost of agent a_i_. The function of LCS is to approach loc_a_i__:

(10) \begin{eqnarray*}\lim _{\mathrm{r}\rightarrow \mathrm{\infty }}\mathrm{es}{\mathrm{t}}_{{\mathrm{c}}_{\mathrm{k}}}^{\mathrm{r}} \left( {\mathrm{d}}_{\mathrm{ i}} \right) =\mathrm{loc}({\mathrm{d}}_{\mathrm{i}})\end{eqnarray*}

We assume that the algorithm converges at the zth round. According to the converse method, we assume that the quality of the algorithm solution decreases as the number of iteration rounds increases. Then the quality of the algorithm solution at round u is less than round u − x, x is the number of rounds in which the algorithm falls into local optimum. To simplify the proof, we assume that *x* = 1. (1 < x < u < z)

(11) \begin{eqnarray*}& {\mathrm{est}}_{{\mathrm{c}}_{\mathrm{k}}}^{\mathrm{u}} \left( {\mathrm{d}}_{\mathrm{ i}} \right) -{\mathrm{est}}_{{\mathrm{c}}_{\mathrm{k}}}^{\mathrm{u}-1} \left( {\mathrm{d}}_{\mathrm{ i}} \right) \lt 0\end{eqnarray*}

(12) \begin{eqnarray*}& {\mathrm{est}}_{{\mathrm{c}}_{\mathrm{k}}}^{\mathrm{u}-1} \left( {\mathrm{d}}_{\mathrm{ i}} \right) \ast {\beta }_{{\mathrm{c}}_{\mathrm{k}}}+{\mathrm{loc}}_{{\mathrm{c}}_{\mathrm{k}},\mathrm{p}}\ast \left( 1-{\beta }_{{\mathrm{c}}_{\mathrm{k}}} \right) -{\mathrm{est}}_{{\mathrm{c}}_{\mathrm{k}}}^{\mathrm{u}-1} \left( {\mathrm{d}}_{\mathrm{ i}} \right) \lt 0\end{eqnarray*}

(13) \begin{eqnarray*}& {\mathrm{loc}}_{{\mathrm{c}}_{\mathrm{k}},\mathrm{p}}\ast \left( 1-{\beta }_{{\mathrm{c}}_{\mathrm{k}}} \right) -{\mathrm{est}}_{{\mathrm{c}}_{\mathrm{k}}}^{\mathrm{u}-1} \left( {\mathrm{d}}_{\mathrm{ i}} \right) \ast \left( 1-{\beta }_{{\mathrm{c}}_{\mathrm{k}}} \right) \lt 0\end{eqnarray*}

(14) \begin{eqnarray*}& ({\mathrm{loc}}_{{\mathrm{c}}_{\mathrm{k}},\mathrm{p}}-{\mathrm{est}}_{{\mathrm{c}}_{\mathrm{k}}}^{\mathrm{u}-1} \left( {\mathrm{d}}_{\mathrm{ i}} \right) )\ast \left( 1-{\beta }_{{\mathrm{c}}_{\mathrm{k}}} \right) \gt 0\end{eqnarray*}

where ${\mathrm{est}}_{{\mathrm{c}}_{\mathrm{k}}}^{\mathrm{u}}$ is is the simulated value of the local cost of the uth round, loc_c_k_,p_ is the local cost of the corresponding round.

Formula (11) is the inequality obtained from the assumption. Bring formula (4) to get formula (12). Merge similar terms to formula (13) and according to formula (10), the value of loc_c_k_,p_ is greater than ${\mathrm{est}}_{{\mathrm{c}}_{\mathrm{k}}}^{\mathrm{u}-1}$, then the assumption does not hold. Therefore, the quality of solutions improves as the number of message rounds increases before convergence.

### Complexity

In this section, we analyze the complexity of LCS. We define the number of agents |A| = n, the number of neighbors of agent x_i_ is |N|.

In the initialization and local cost simulation, only population messages need to be sent per round, so the size of the message is *O*(|C|∗|P|), where |C| is the number of populations and |P| is the number of individuals per population.

The time complexity of LCS mainly depends on the update of simulations and the calculation of value messages. When updating the simulations, it is necessary to calculate the local cost for each individual. When calculating local cost, one individual requires *O*(|N|) complexity. So, complexity for all populations requires *O*(|C|∗|P|∗|N|). For the calculation of the value message, the agent just needs to traverse the simulations to calculate the probability. The calculation of probability and selection of value can be done sequentially. So, the time complexity of the calculation of the value message is *O*(|C|∗max(|P|, |M|)), where |M| is the length of the domain. In summary, the time complexity of LCS is *O*(n).

## Empirical Evaluations

### Benchmark problems

We empirically evaluate our proposed method with peer algorithms including DSA (type-C and *p* = 0.6), MGM, GDBA, PDS, ACO_DCOP ( *K* = 19), Max-sum_ADVP, Damped Max-sum and LSGA. DSA, MGM and GDBA are implemented under the ALS framework. For PDS and LSGA, we choose PDS-DSA and LSGA-DSA (*M* = 18) who perform the best. And LCS is run on ALS framework to record the optimal solution during the iterations.

Our extensive empirical evaluations are benchmarked on four types of problems including random DCOPs, scale-free networks (SFN), weighted graph coloring problems (WGC) and random meeting scheduling problems (Chen et al., 2020). The recommended values of *α* are shown in Table 4. For random DCOPs, we set the agent number to 70 and 120, domain size to 10, choose the constraint uniformly from [1,100], and consider the graph density 0.1 (for sparse) or 0.6 (for dense). For SFN, we set the agent number to 150, domain size to 10, choose the constraint uniformly from [1,100], and consider the problems with m_1_ = 20, m_2_ = 3 (for sparse) and m_1_ = 20, m_2_ = 10 (for dense). For WGC, we set the agent number to 120, the available color number to 3, constraint density to 0.05, and choose the constraint uniformly from [1,100]. For random meeting scheduling problems, we set the agent number to 90, the meeting number to 20, the available time-slots to 20, and travel times are randomly selected from 6 to 10. The experimental results take the average of 50 independent problems that each execute 30 times. We run our experiments on a laptop with an Intel Core i7-6700 CPU 2.60 GHz and 8 GB RAM.

### Memory footprints & network load

In terms of memory footprints, we set different population sizes to test on random DCOPs and scale-free networks. We take the maximum value of memory footprints for multiple runs of the algorithm. Table 5 shows that population sizes, density and the agent number are all proportional to memory footprints. In terms of network load (Nguyen et al., 2019), that is, the amount of information passed around the network, LCS send a polynomial amount of information in each iteration.

### Influence of population size

In order to discuss the influence of population size on the performance of the LCS, we ensure that the parameters *α*, *β*, *γ* of LCS are constant and use many different combinations of population sizes of C = 1, 2, 3, 4, 5, *P* = 8, 16, 24, 30. We choose sparse random graphs of 70 agents and the obtained results are shown in Table 6. From the analysis of the results, the higher the number of populations C and the number of individuals P is set, the higher the solution quality of the algorithm is, and the running time of the algorithm increases. However, after the solution quality decreases to a certain level, continuing to increase C and P only results in a small gain. At this point, the increasing trend of the algorithm running time becomes larger and the gain ratio obtained is not high. Therefore, according to the results of the table, C = 4, P = 24 is chosen to weigh the solution quality and running time.

**Table 4:** The recommended value for *α*.

Problems	*α*
DCOP_70_0.1	11
DCOP_70_ 0.6	32
DCOP_120_0.1	15
DCOP_120_0.6	43
SFN_150_20_3	10
SFN_150_20_10	20
WGC_120_0.05	2
Meeting scheduling	14

**Table 5:** The memory footprints of LCS with different population sizes on random DCOPs and SFN (memory footprints(MB)).

C*P	random DCOPs				SFN	
	A=70		A=120		A=150	
	sparse	dense	sparse	dense	sparse	dense
4*8	66.9	165.7	102.5	500.1	154.8	403.8
4*16	68.7	172.8	107.4	539.4	162.9	466.5
4*24	68.9	174.2	122.6	572.6	166.9	498.3
4*30	69.1	190.2	142.6	583.2	175.0	589.6

**Table 6:** Comparison of different C and P on random DCOPs (|A| = 70, *p* = 0.1) (cost/time (ms)).

P/ C	8	16	24	30
1	5970/858	5481/896	5405/924	5383/932
2	5416/894	5327/949	5300/947	5289/951
3	5331/899	5277/983	5258/1001	5250/1077
4	5287/917	5261/995	5240/1093	5234/1197
5	5269/930	5250/1077	5232/1231	5228/1441

### Influence of population learning rate

In order to discuss the influence of population learning rate on the performance of the LCS, we set different population learning rate *γ* and the obtained results are shown in Table 7. We still choose sparse random graphs of 70 agents and *γ* = 0, 0.1, 0.3, 0.5, 0.7, 0.9. And to make the population emphasize different phases, *β* = 0.9, 0.8, 0.7, 0.6 are selected, but you can also try other values. By analyzing the experimental results, when *γ* = 0, no value exchange between populations was performed at this time. Comparison with the cost of the algorithm after the introduction of the population learning rate *γ* shows poor results. The effectiveness of the algorithm was improved after the introduction of *γ*, which confirmed the effectiveness of population cooperation. According to the result, *γ* = 0.7 performs the best solution.

**Table 7:** Comparison of different on random DCOPs (|A| = 70, *p* = 0.1).

*γ*	0	0.1	0.3	0.5	0.7	0.9
cost	5302	5270	5262	5249	5240	5245

### Influence of population cooperation mechanism

In this section, we evaluate the influence of the population cooperation mechanism. We call the LCS without population cooperation mechanism as LCS-WPCM. We set the same parameter for both algorithms as $\mathrm{C}=4, \mathrm{P}=24, \beta = \left\{ 0.9,0.8,0.7,0.6 \right\} $ and *γ* = 0.7.

**Figure 2:**
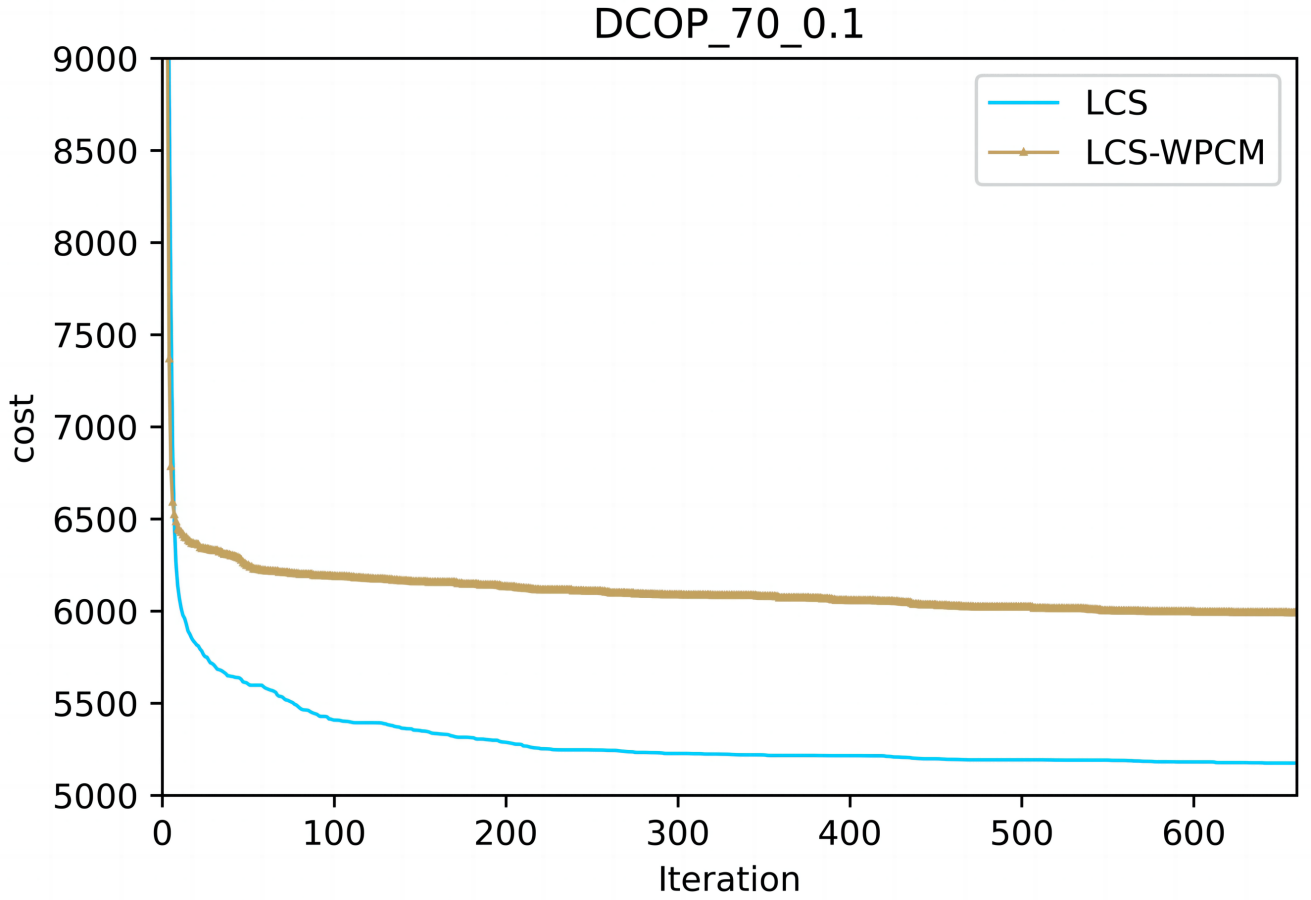
Sparse random graphs (|A|= 70, *p* = 0.1).

**Figure 3:**
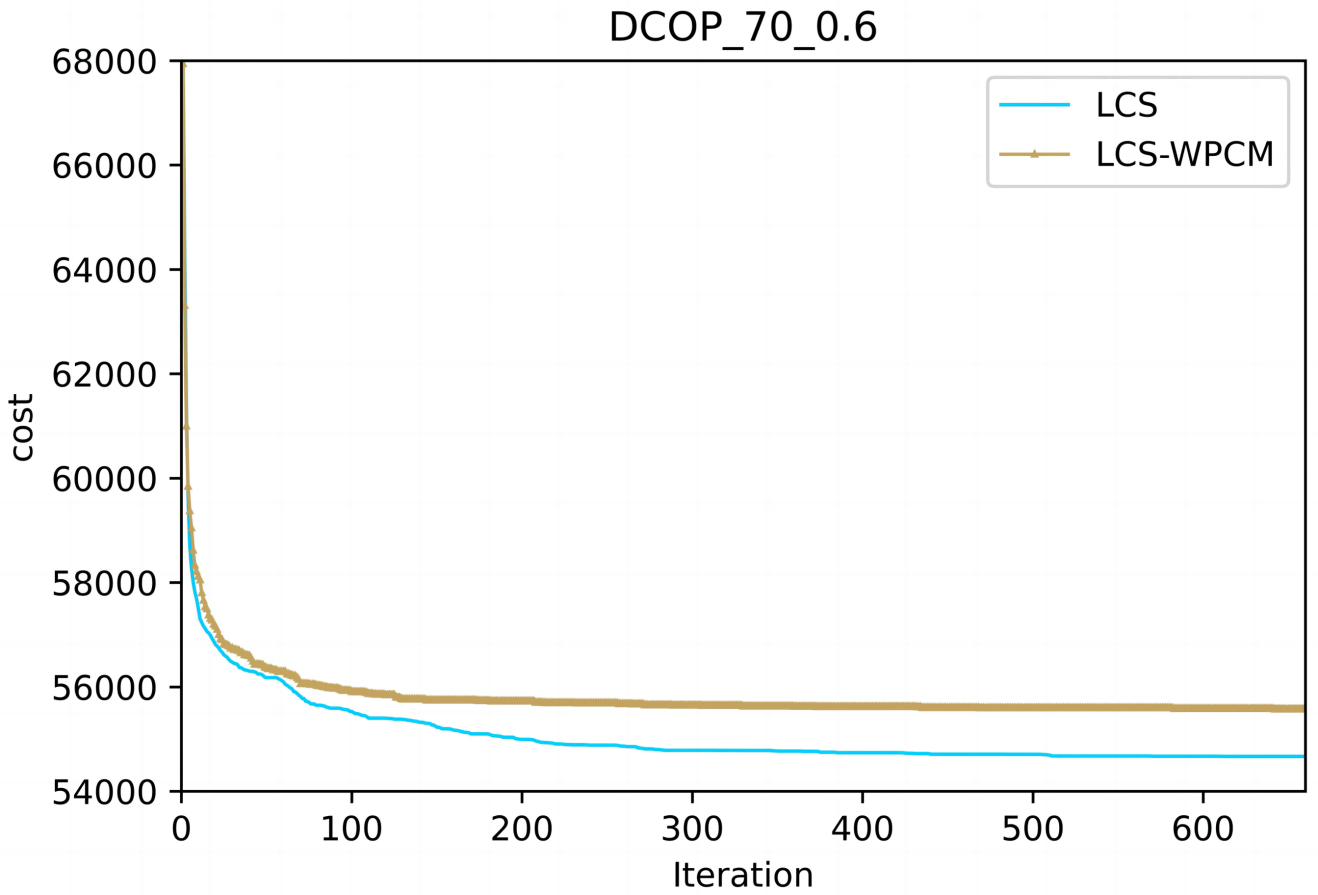
Dense random graphs (|A|= 70, *p* = 0.6).

Figures 2 and 3 show the convergence curve of LCS-WPCM and LCS on sparse and dense random graphs with 70 agents. From Figs. 2 and 3, we can see that whether in sparse graph or dense graph, population cooperation mechanism can improve the convergence quality of LCS.

### Comparisons with the state-of-the-art algorithms

In this section, LCS was compared with the comparison algorithm on the four Benchmark problems. According to the previous analysis, we set the parameters of LCS as $\mathrm{C}=4, \mathrm{P}=24, \beta = \left\{ 0.9,0.8,0.7,0.6 \right\} $ and *γ* = 0.7. The Max-sum_ADVP and the Damped Max-sum for our comparison experiments are non-anytime, so we give the experimental results separately.

**Figure 4:**
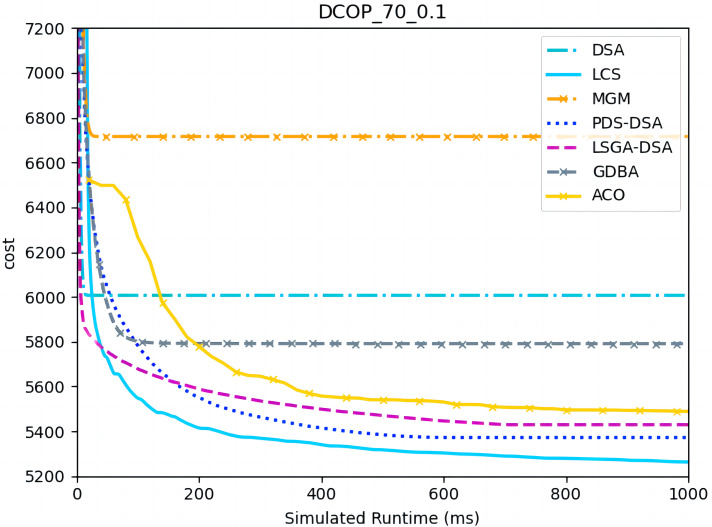
The cost of LCS, DSA, MGM, GDBA, PDS-DSA, ACO and LSGA-DSA for random DCOPs (|A|= 70, *p* = 0.1).

**Figure 5:**
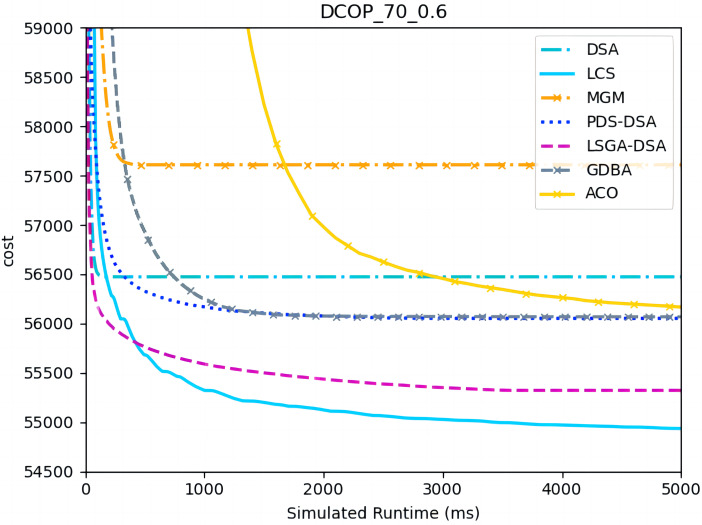
The cost of LCS, DSA, MGM, GDBA, PDS-DSA, ACO and LSGA-DSA for random DCOPs (|A|= 70, *p* = 0.6).

**Figure 6:**
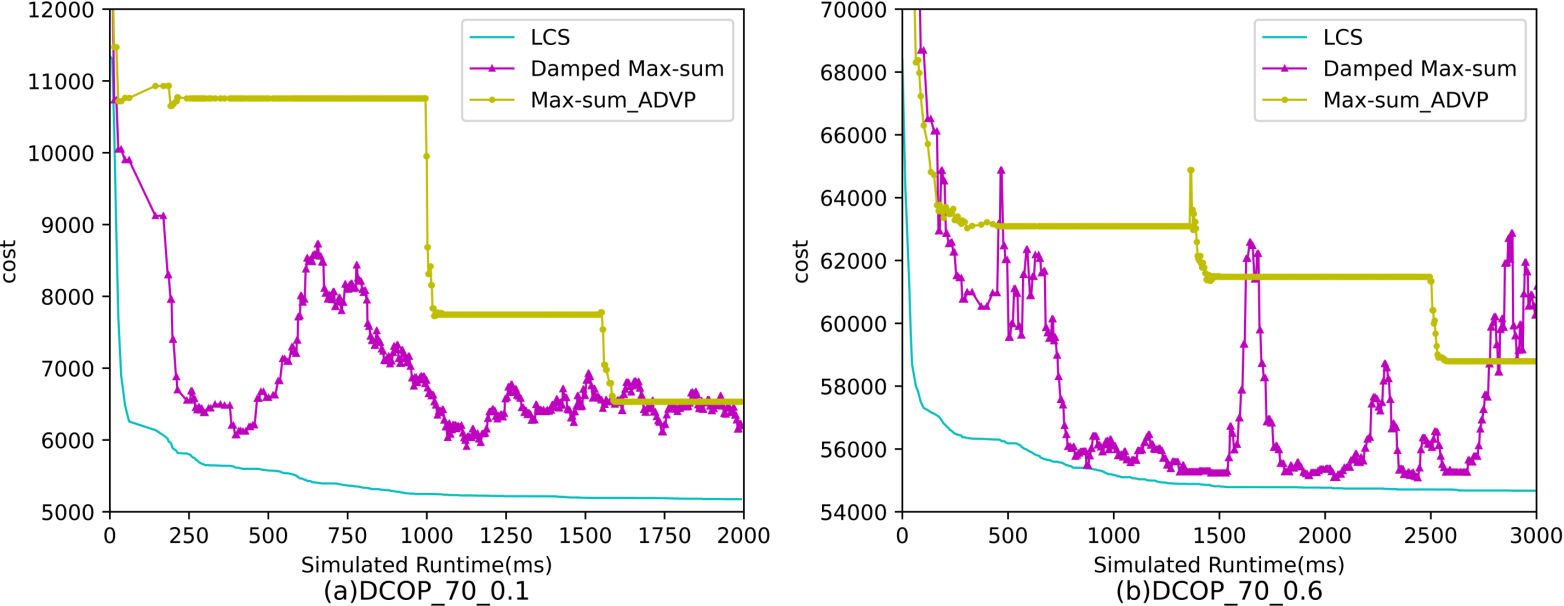
The cost of LCS, Max-sum_ADVP and Damped Max-sum for random DCOPs (|A|= 70).

Figures 4–6 show the comparison between LCS and other algorithms in random DCOPs (|A| = 70, p = 0.1; |A| = 70, p = 0.6), and Table 8 gives the comparisons of all other problems. All differences are statistically significant for p − value < 1 × 10^−36^. Compared to the other algorithms, LCS improves the solution quality on sparse problems by 2.2% ∼ 21.8% and on dense problems by 0.7% ∼ 5.7%. By analyzing the decline curve of LCS, it can be found that some more twists and turns are caused by multiple search phases and population interaction. In the initial phase of iteration, LCS decreases more slowly than LSGA-DSA or ACO_DCOP because estimations have not been simulated well. But after a full search, LCS performs better than all other algorithms and cost shows a slow downward trend all the time. LCS has good search ability for both sparse and dense problems, while the other algorithms are biased to only one of them and cannot catch up with the LCS algorithm.

**Figure 7:**
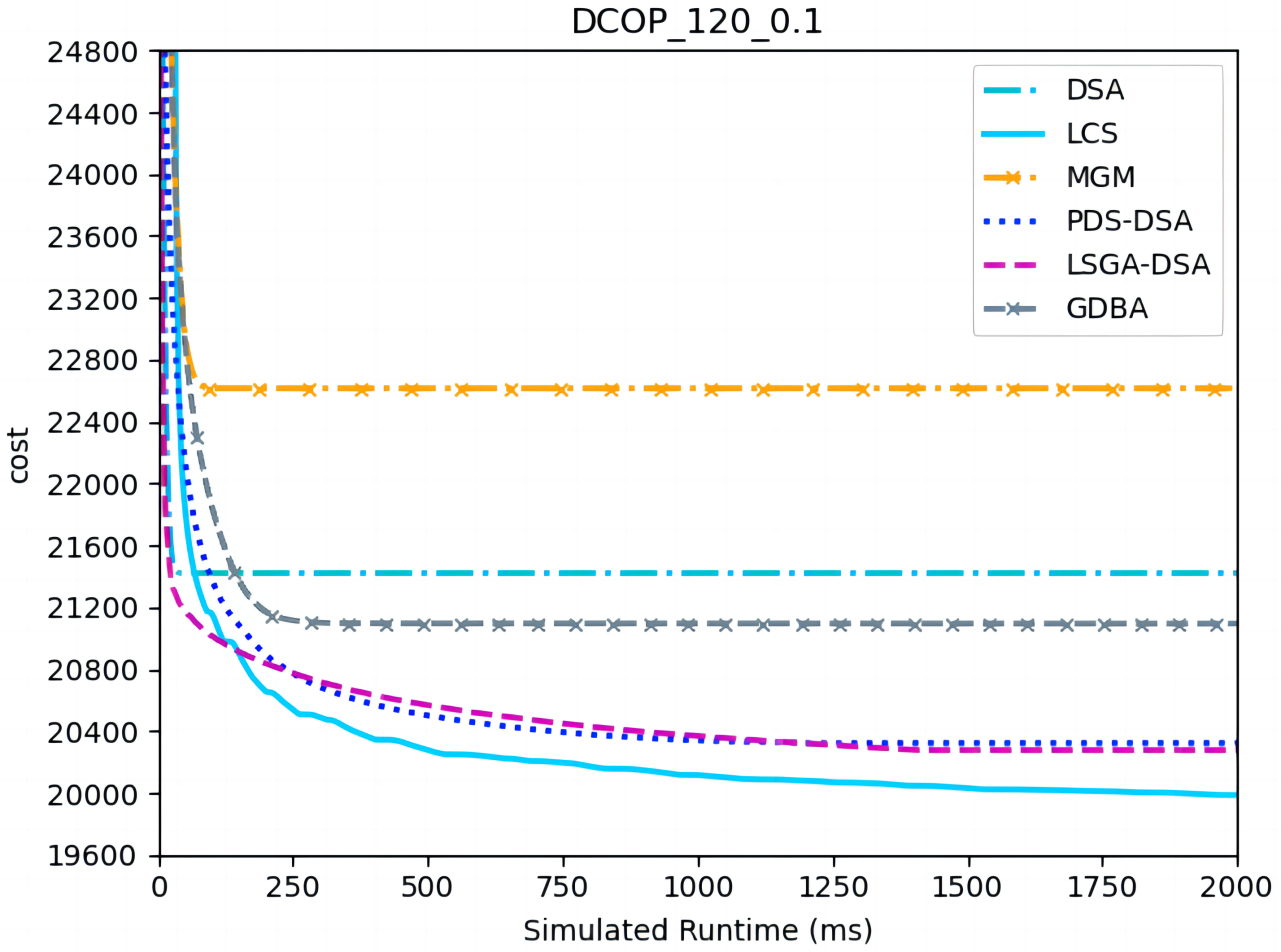
The cost of LCS, DSA, MGM, GDBA, PDS-DSA and LSGA-DSA for random DCOPs (|A|= 120, *p* = 0.1).

**Figure 8:**
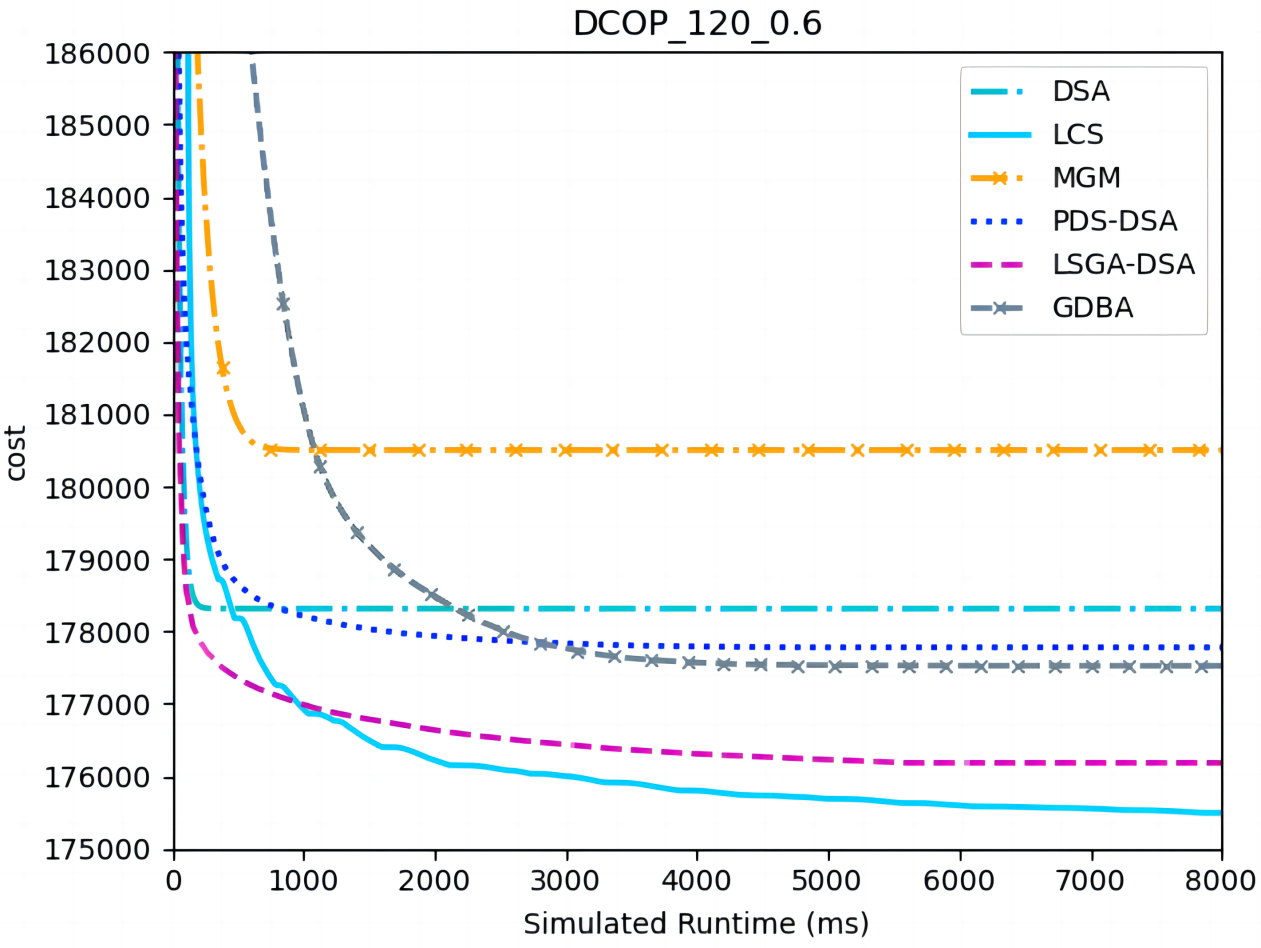
The cost of LCS, DSA, MGM, GDBA, PDS-DSA and LSGA-DSA for random DCOPs (|A|= 120, *p* = 0.6).

**Figure 9:**
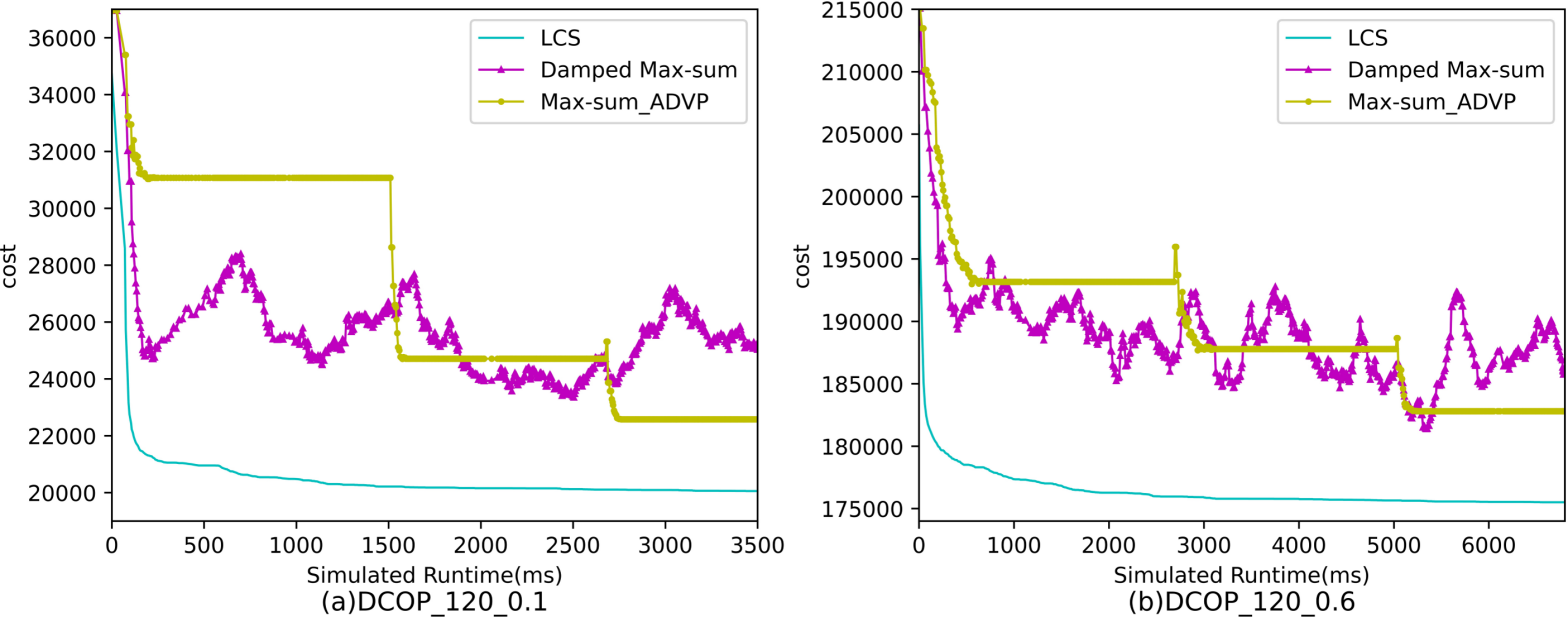
The cost of LCS, Max-sum_ADVP and Damped Max-sum for random DCOPs (|A|= 120).

Figures 7–9 show the comparison between LCS and other algorithms in random DCOPs (|A| = 120, p = 0.1; |A| = 120, p = 0.6). Compared to the other algorithms, LCS improves the solution quality on sparse problems by 1.5% ∼ 19.3% and on dense problems by 0.4% ∼ 4.0%. Population-based LSGA-DSA and LCS with population achieved better results than other algorithms, but LSGA-DSA results were not as good as LCS, which proves that the local cost of simulation in DCOP is better than genetic algorithm coding. Since the global cost increases with the number of agents and the problem size, the degree of improvement of LCS is lower than before. However, the LCS algorithm still has higher solution quality than other algorithms for sparse and dense problems due to the local cost simulation method and population cooperation with each other.

**Figure 10:**
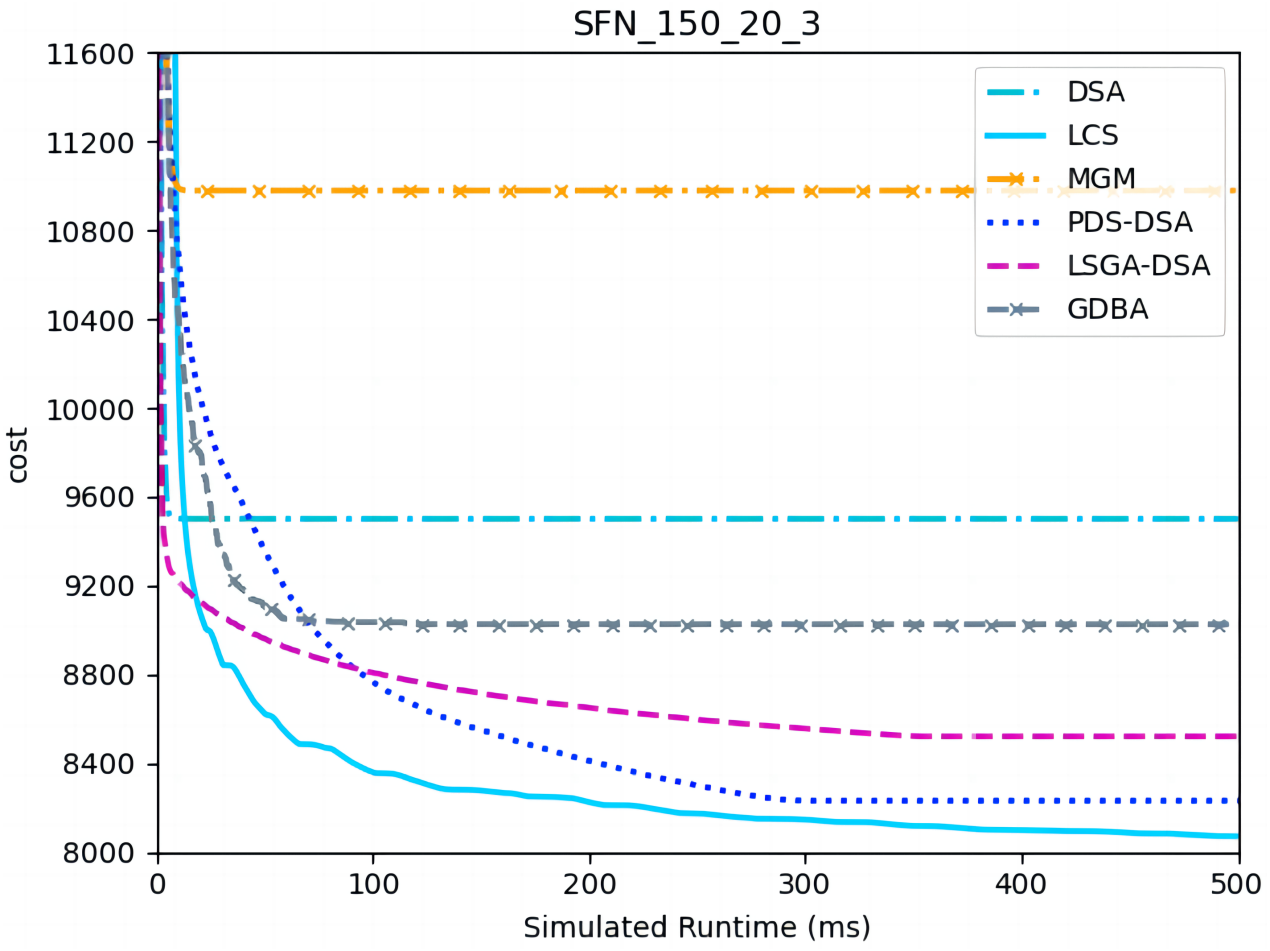
The cost of LCS, DSA, MGM, GDBA, PDS-DSA and LSGA-DSA for scale-free networks (|A|= 150, m1 = 20, m2 = 3).

**Figure 11:**
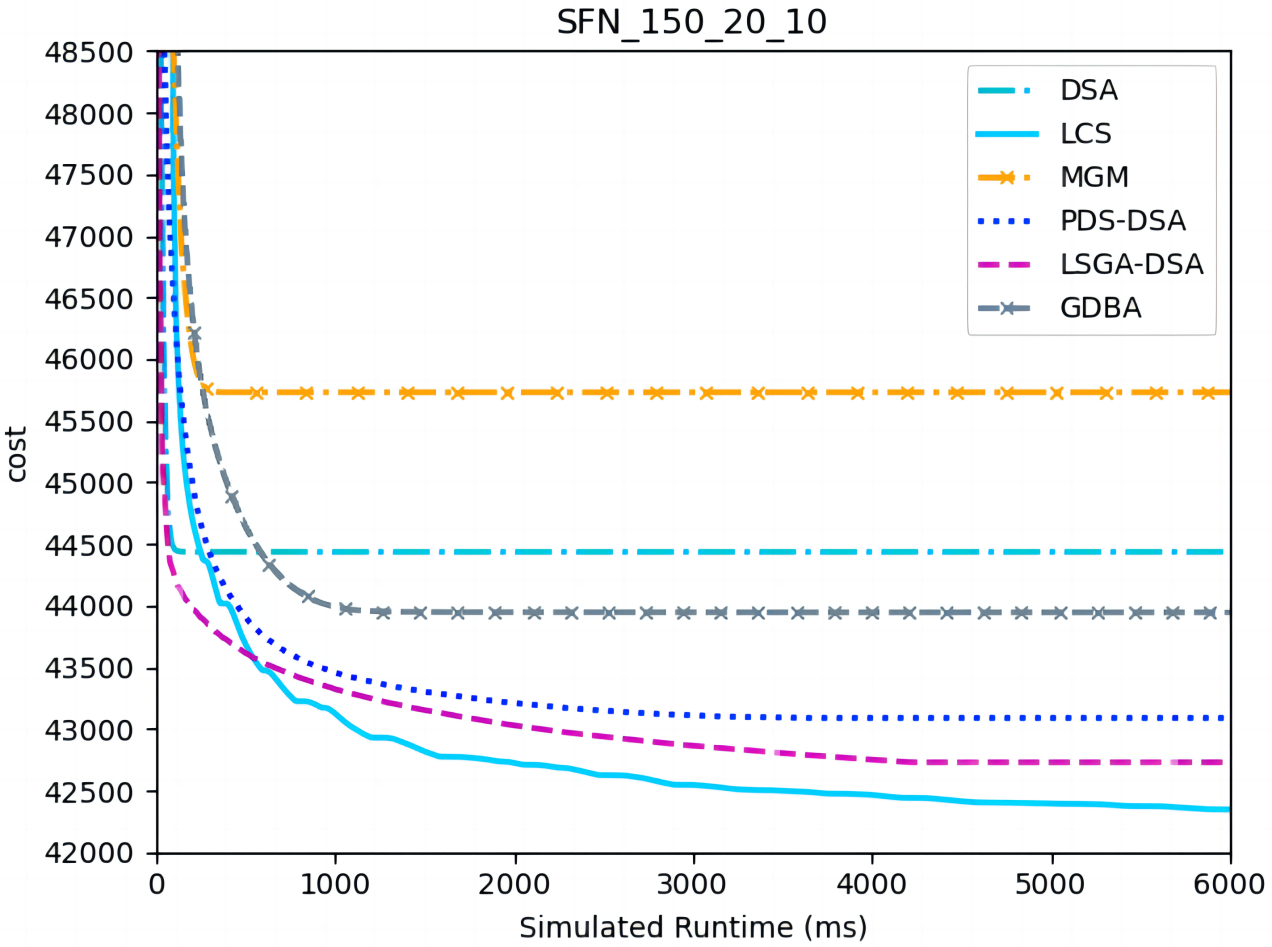
The cost of LCS, DSA, MGM, GDBA, PDS-DSA and LSGA-DSA for scale-free networks (|A|= 150, m1 = 20, m2 = 10).

**Figure 12:**
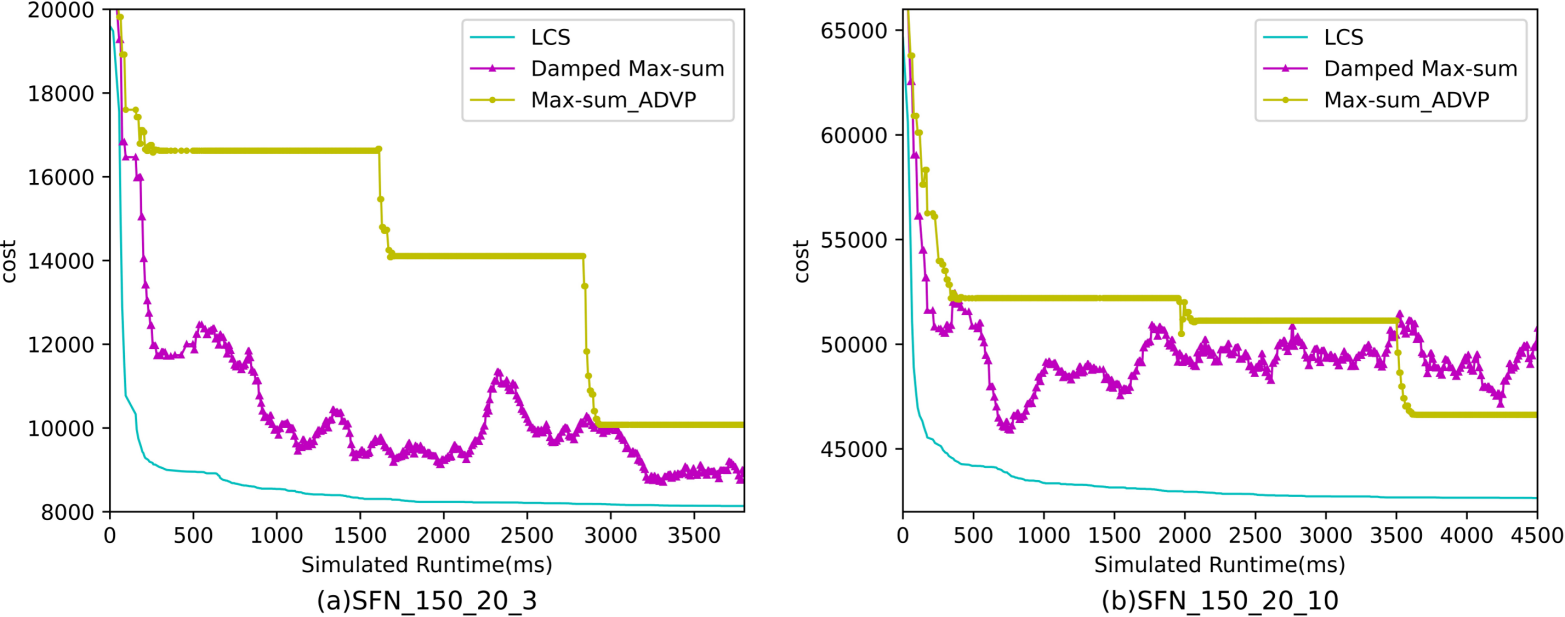
The cost of LCS, Max-sum_ADVP and Damped Max-sum for scale-free networks (|A|= 150).

Figures 10– 12 show the comparison between LCS and other algorithms in scale-free networks( |A| = 150, m1 = 20, m2 = 3; |A| = 150, m1 = 20, m2 =10). From the experimental results it can be seen that the algorithm runs longer on this problem because of its larger size. Compared to the other algorithms, LCS improves the solution quality on sparse problems by 2.1% ∼ 26.6% and on dense problems by 1.0% ∼ 9.8%. The improvement effect shows that the algorithm improves less on the dense problem than on the sparse problem. The reason for this is that as the number of neighbors connected to the centroids in the scale-free network increases, the simulation accuracy of the local cost is affected and its fluctuation range becomes larger. However, the algorithm preserves the probability of being selected for all values in the value domain, so the effect of this error is improved, which makes the LCS still outperform the other algorithms. Table 8 shows that LCS has good search capability for both sparse and dense problems in different problems, where other algorithms can not catch up with the effect of LCS.

**Table 8:** Comparison of LCS and the benchmarking algorithms on difference configuration of random DCOPs and SFN.

Problems	random DCOPs				SFN	
	A=70		A=120		A=150	
	sparse	dense	sparse	dense	sparse	dense
DSA	6006	56473	21421	178316	9500	44435
MGM	6715	57609	22615	180505	10978	45729
PDS-DSA	5372	56052	20322	177781	8231	43088
LSGA-DSA	5429	55321	20275	176185	8521	42728
GDBA	5791	56069	21093	177529	9025	43944
ACO_DCOP	5436	55790	–	–	–	–
Max-sum_ADVP	6574	58272	21808	182814	9818	46963
Damped Max-sum	6019	55356	24766	181156	8287	46572
LCS	5257	54922	19970	175461	8059	42322

**Figure 13:**
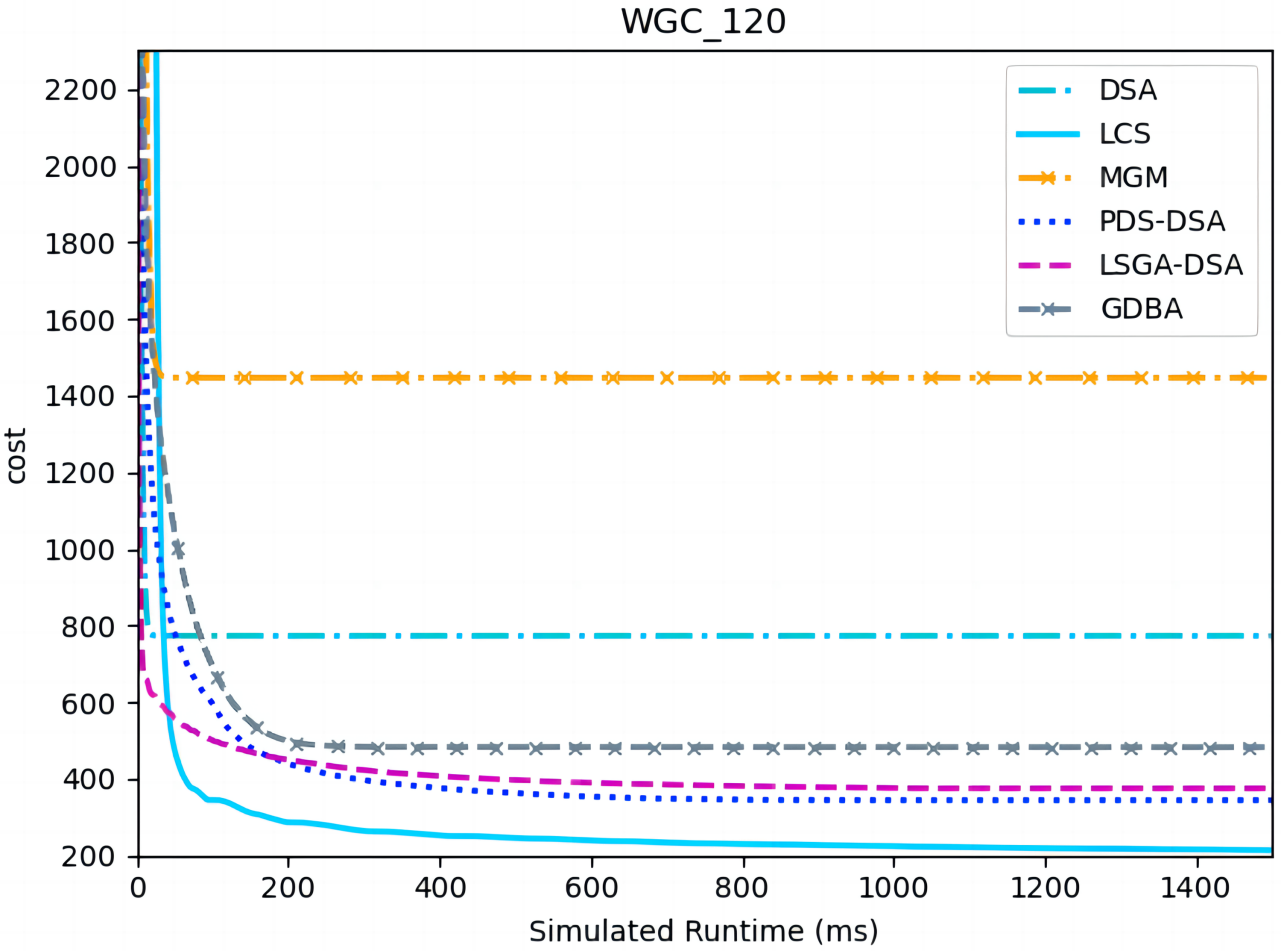
The cost of LCS, DSA, MGM, GDBA, PDS-DSA and LSGA-DSA for weighted graph coloring problems with 120 agents.

**Figure 14:**
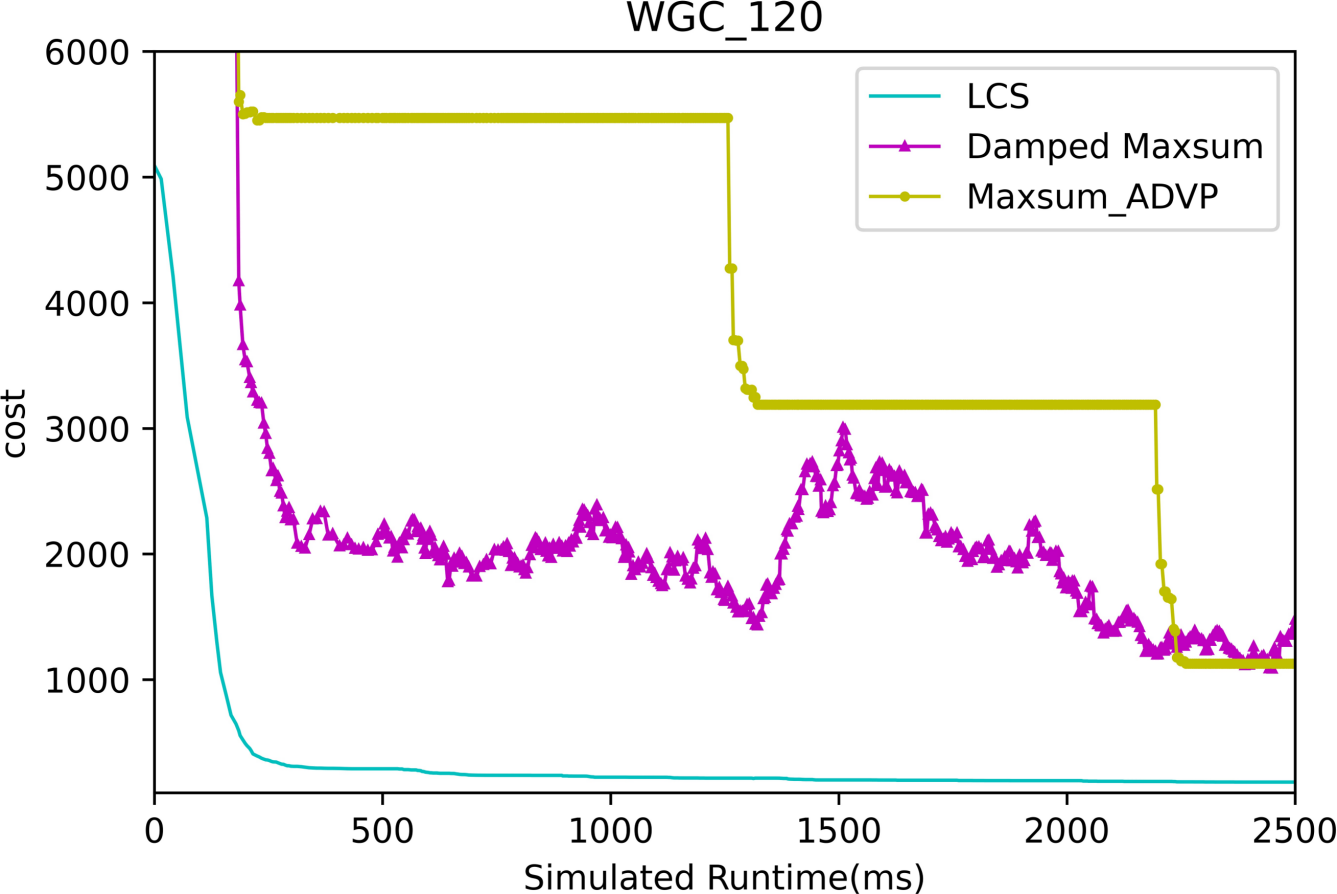
The cost of LCS, Max-sum_ADVP and Damped Max-sum for weighted graph coloring problems with 120 agents.

Figures 13 and 14 presents the comparison of LCS with other algorithms on weighted graph coloring problems (|A| = 120, p = 0.05). Compared to the other algorithms, LCS improves by 38.4% ∼ 85.3%, which is a great improvement. In this problem, it can be found that all algorithms solve with a lower value of global cost, and the solution of PDS-DSA algorithm has dropped to more than 300, which is already a better solution. However, in contrast to it, LCS drops the solution to about 200, which greatly improves the solution quality. The weighted graph coloring problem favors DCSP (Yokoo et al., 1998) similarly, which proves that LCS may be superior in solving similar problems such as DCSP.

**Figure 15:**
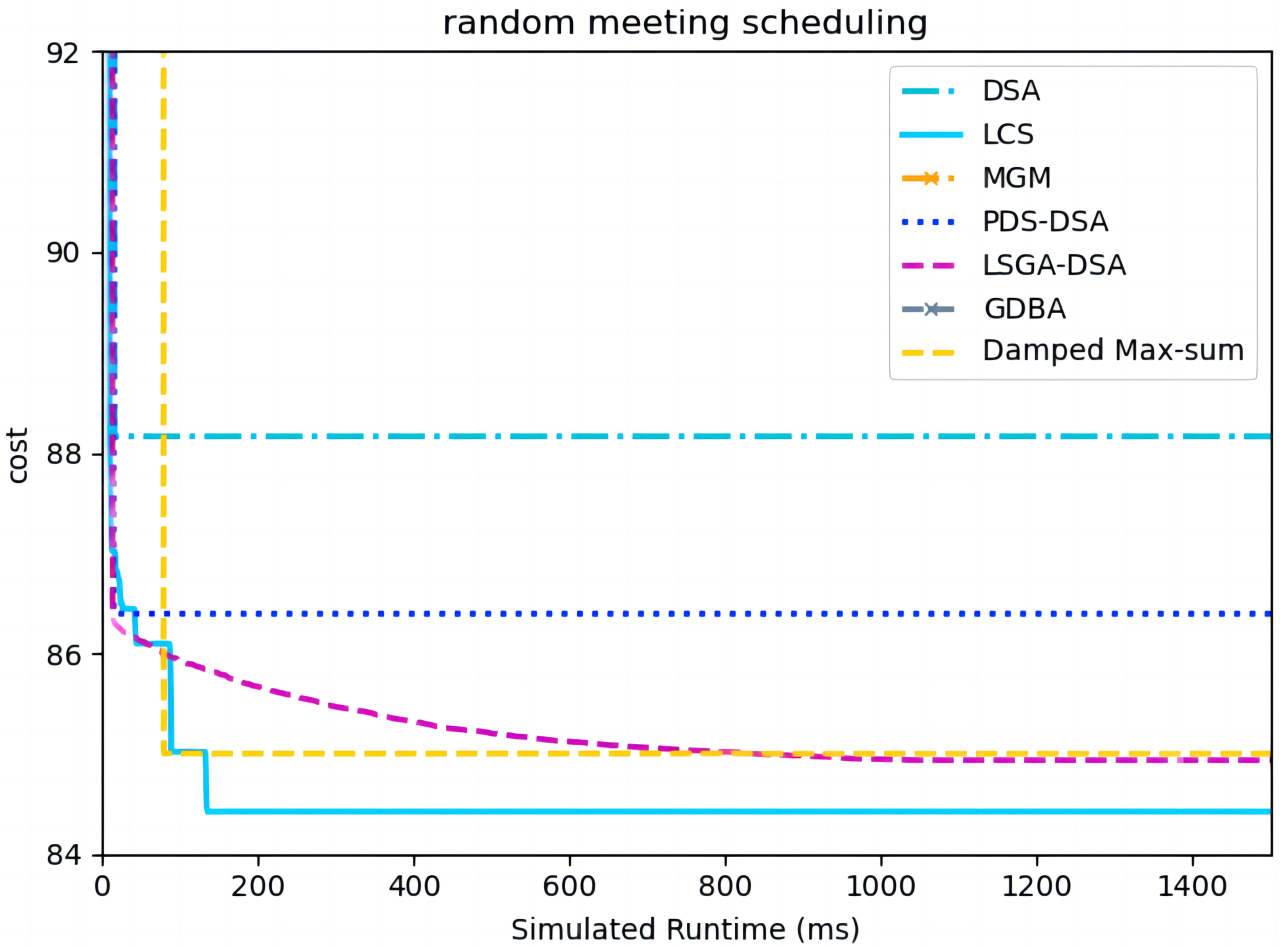
The cost of all algorithms for random meeting scheduling problems with 90 agents.

Figure 15 presents the comparison of LCS with other algorithms on random meeting scheduling problems(|A| = 90). We omit the results of MGM and GDBA due to their inferior performances (Chen et al., 2020). Compared to the other algorithms, LCS improves by 0.6% ∼ 4.2%. In this problem, it can be found that all algorithms solve with a lower value of global cost. Experimental results show that LCS outperforms other algorithms and can improve real-world problems.

## Conclusions

The changing of the local cost that can be obtained from historical agent values has a significant influence on the performance of the local search algorithm. LCS is presented in this article to exploit the potential of historical values of agents to further develop the exploration ability of the local search algorithm. The proposed LCS makes up for the vacancy that local cost historical information has never been considered in the local search algorithms. In LCS, the designed EWMA provides an effective scheme for estimating local cost and the population interact mechanism giving a strategy to improve the exploitation and exploration ability of local search algorithms. We theoretically analyze the superiority of LCS from two different aspects. Finally, our experimental results show that LCS is superior to the competing algorithms including the latest local search algorithms and population-based algorithms. In future work, we will explore how to solve the problem of reliance on parameter *α* and make LCS self-adaptive according to the scale of the problem.

##  Supplemental Information

10.7717/peerj-cs.1296/supp-1 Supplemental Information 1 Code

10.7717/peerj-cs.1296/supp-2 Supplemental Information 2 Supplemental Figures

## Additional Information and Declarations

### Data Availability

The following information was supplied regarding data availability: The code is available in the Supplementary File.
