# Supplementary figures and images for: A local cost simulation-based algorithm to solve distributed constraint optimization problems
Source: PeerJ Comput Sci. 2023 Mar 17;9:e1296. doi: 10.7717/peerj-cs.1296 (PMC10280401; doi:10.7717/peerj-cs.1296)

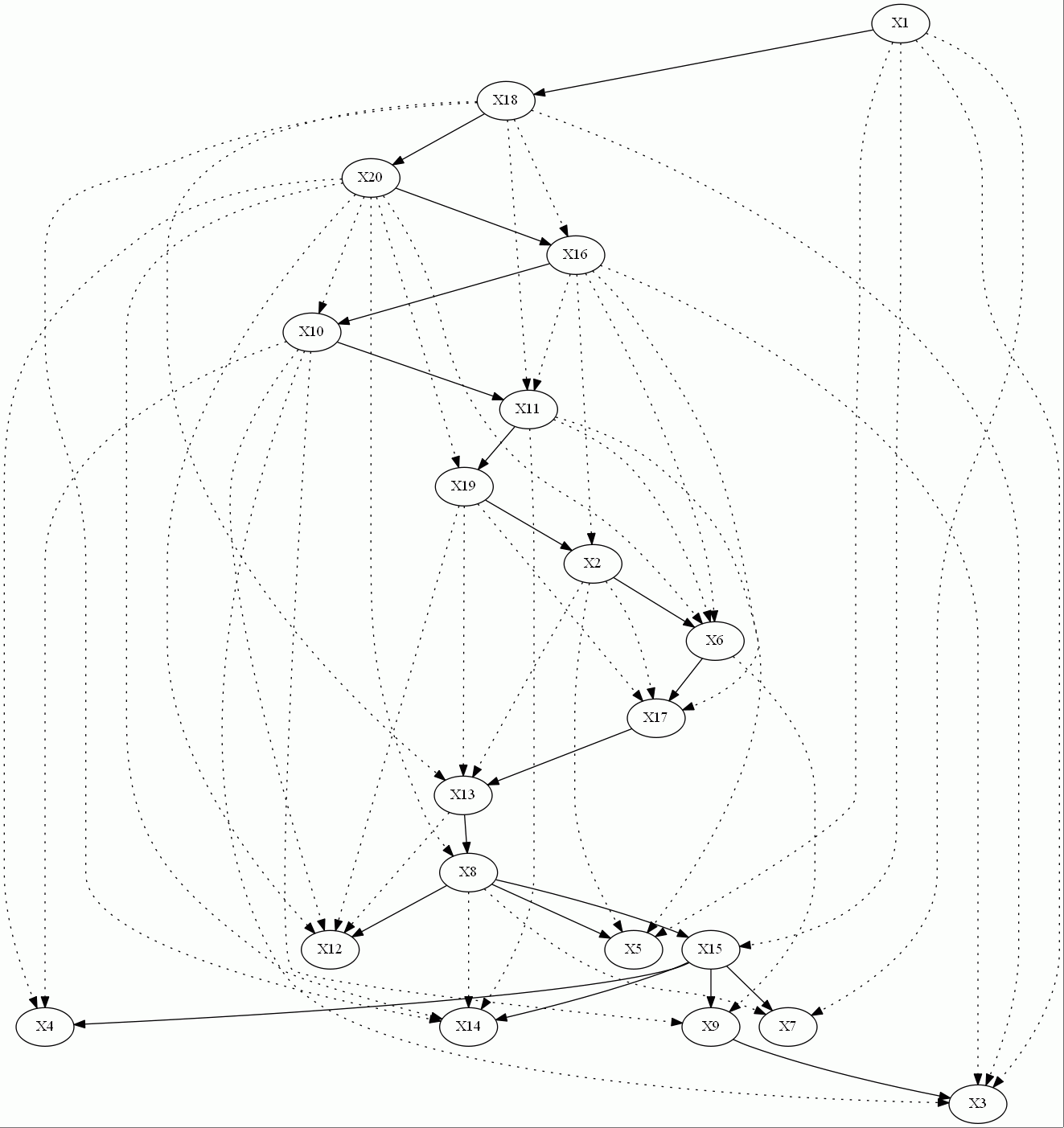

Supplement: Supplemental Information 1 [file peerj-cs-09-1296-s001.zip › code/DCOP_LCS/1.PNG]

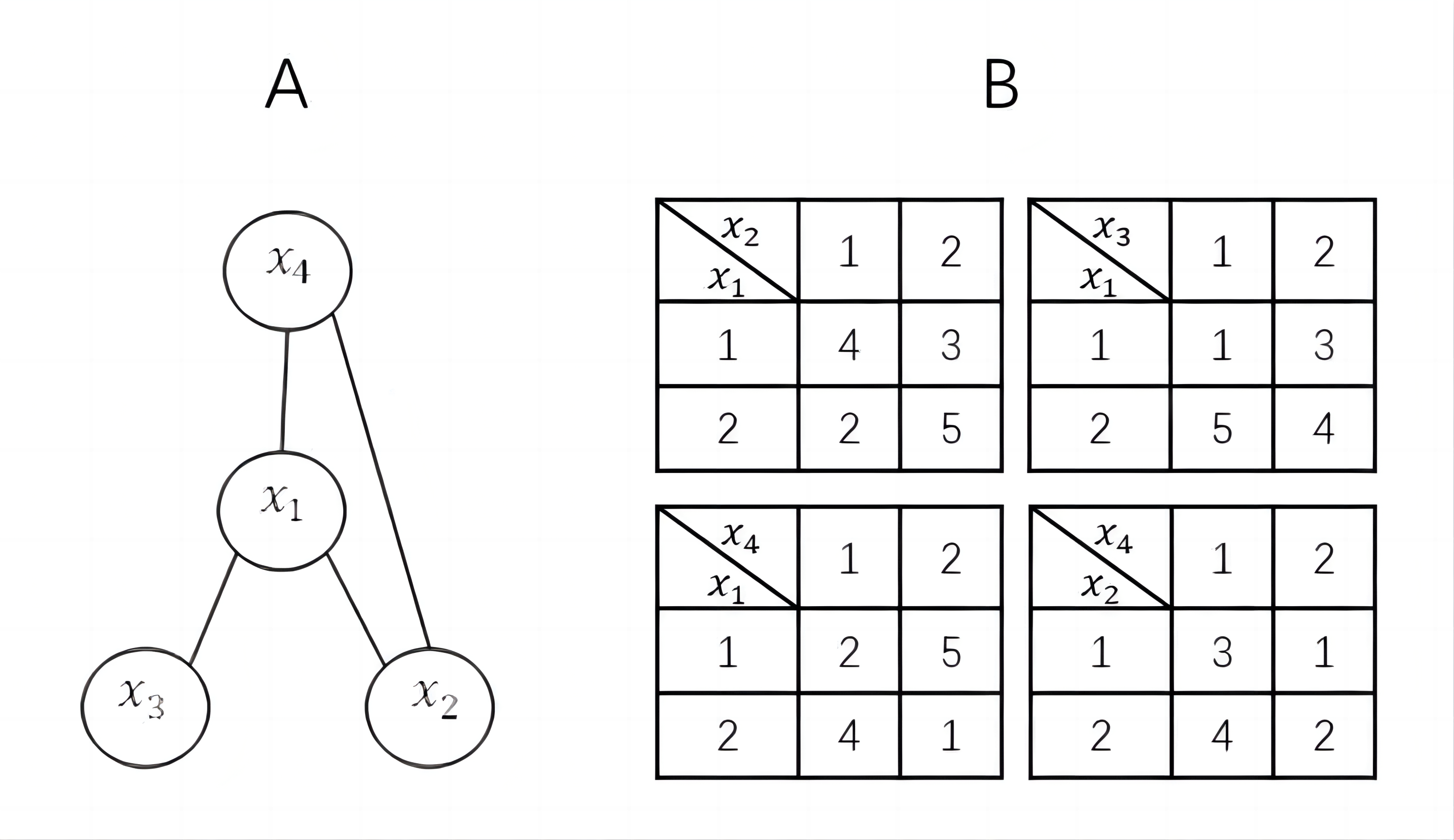

Supplement: Supplemental Information 2 [file peerj-cs-09-1296-s002.zip › Supplemental Figures S2/Figure1.png]

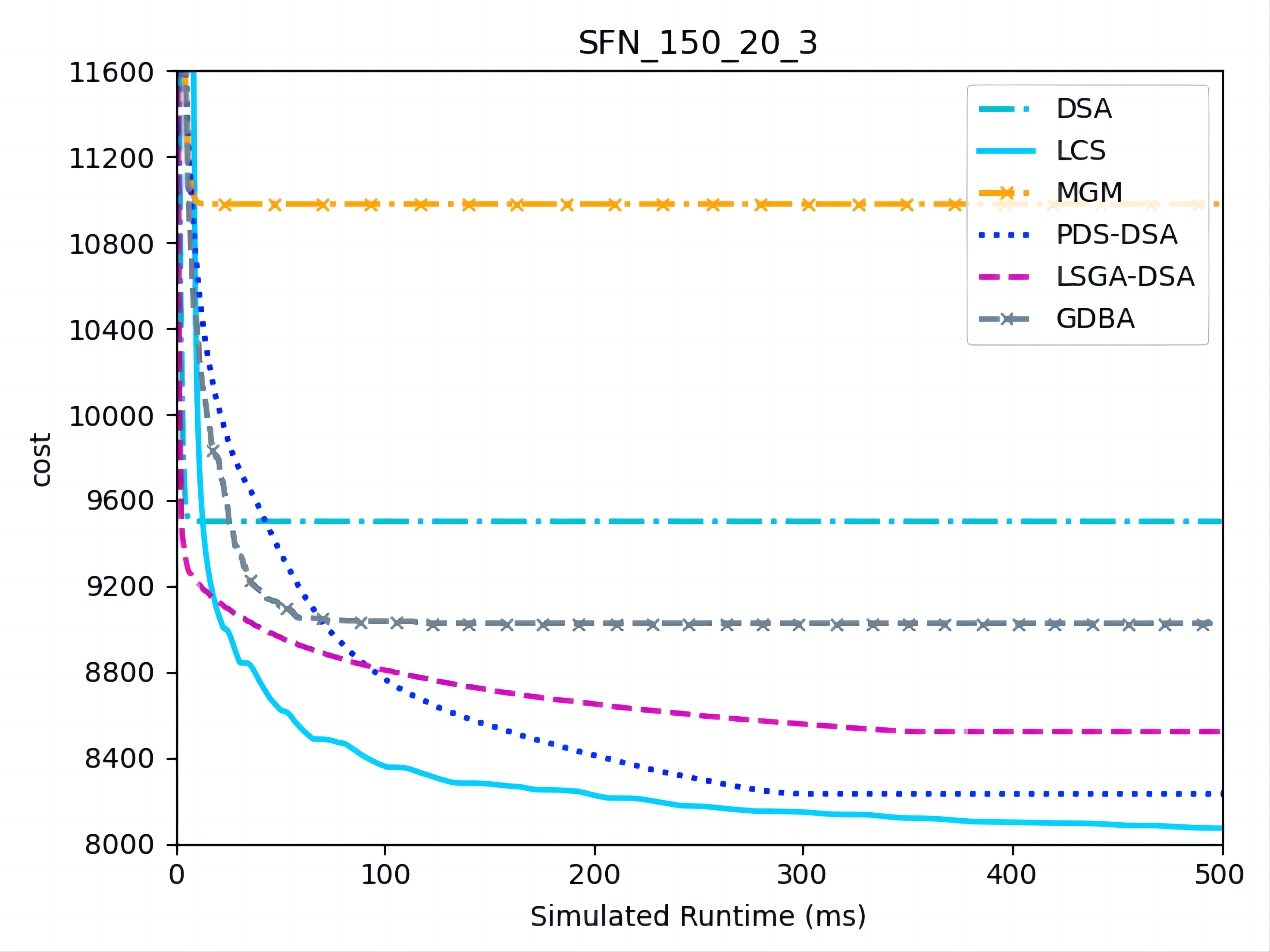

Supplement: Supplemental Information 2 [file peerj-cs-09-1296-s002.zip › Supplemental Figures S2/Figure10.png]

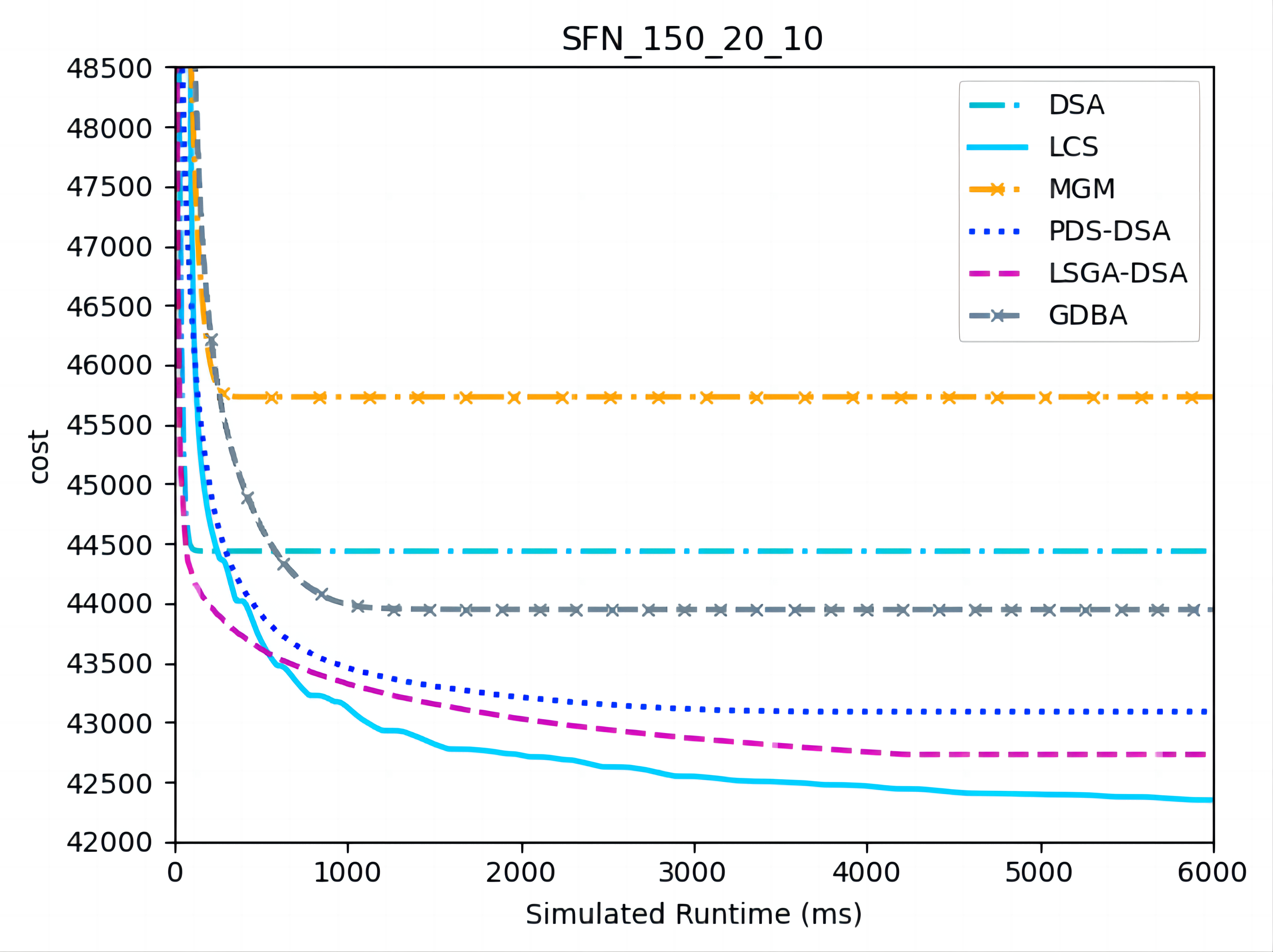

Supplement: Supplemental Information 2 [file peerj-cs-09-1296-s002.zip › Supplemental Figures S2/Figure11.png]

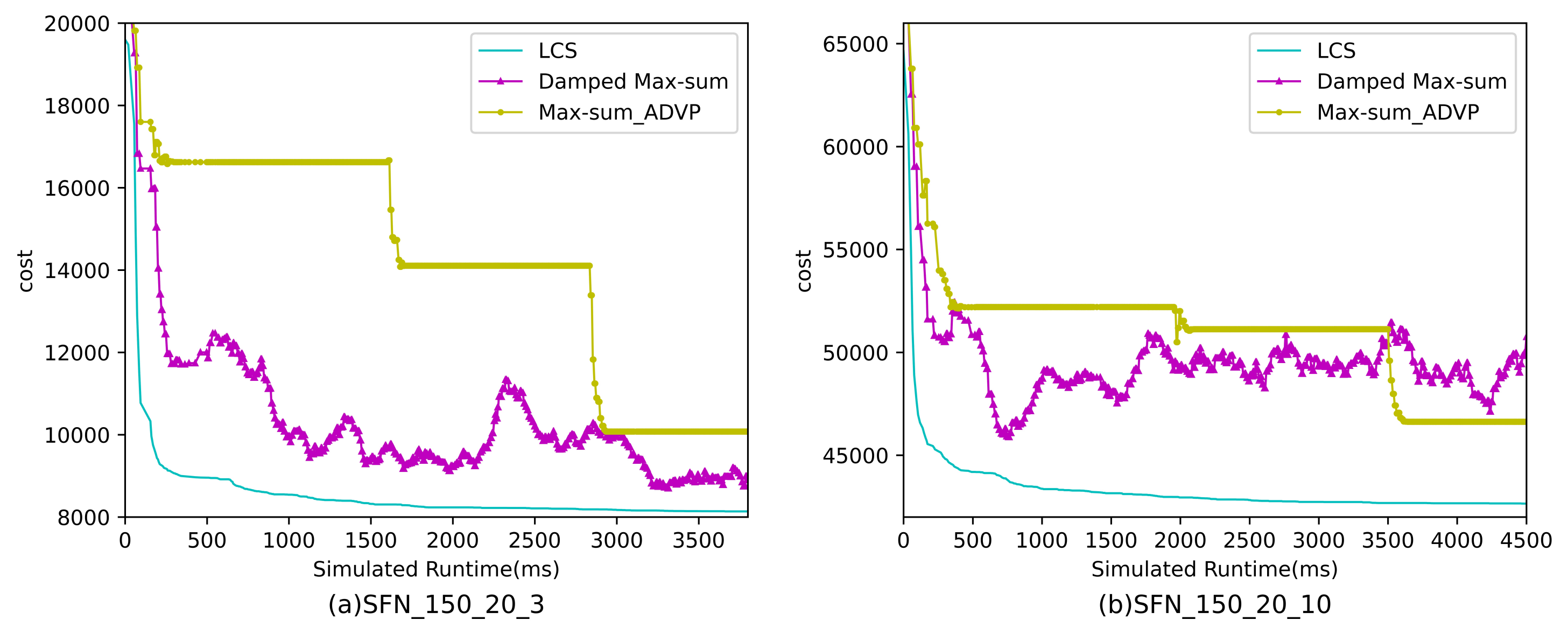

Supplement: Supplemental Information 2 [file peerj-cs-09-1296-s002.zip › Supplemental Figures S2/Figure12.png]

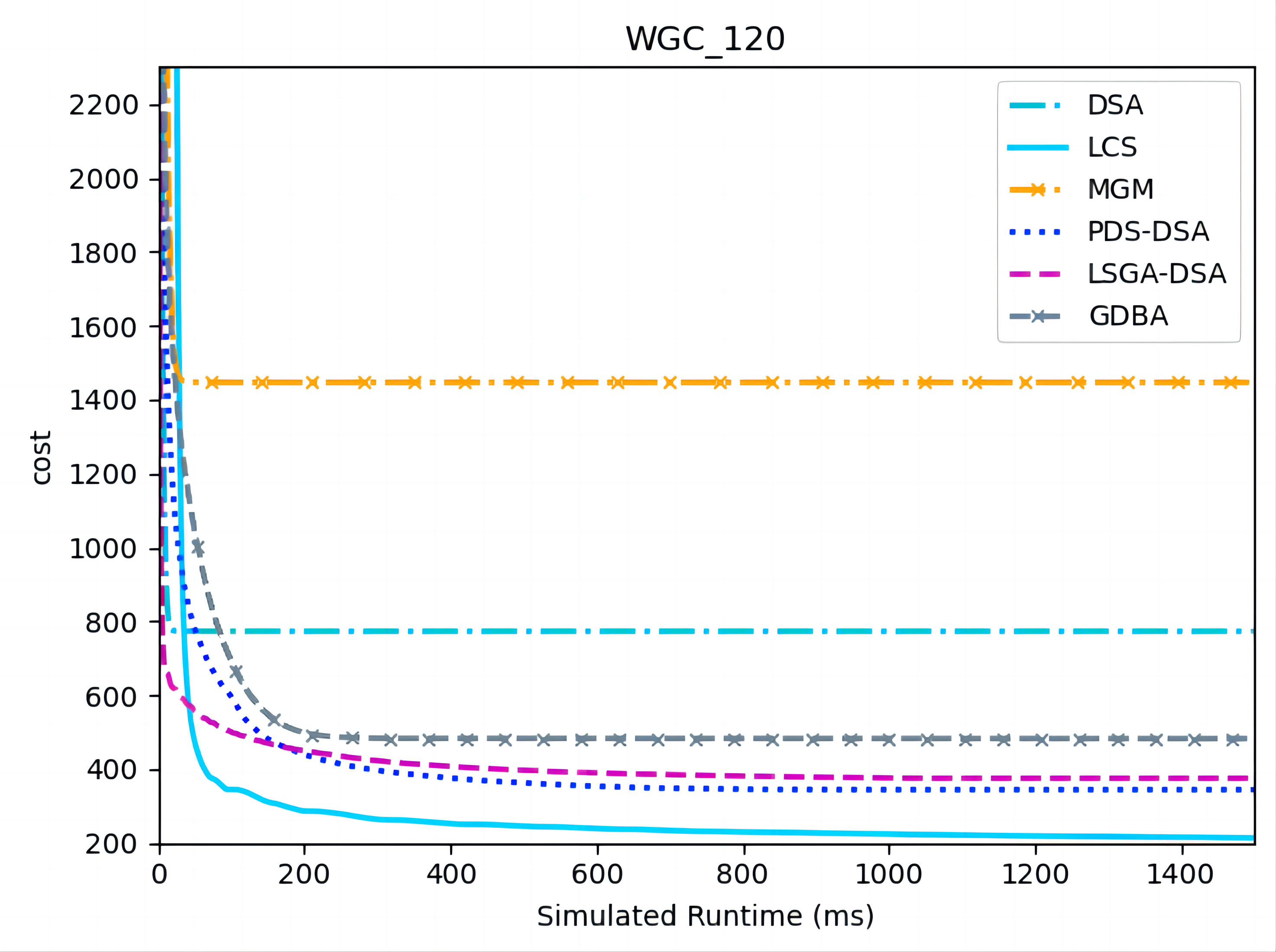

Supplement: Supplemental Information 2 [file peerj-cs-09-1296-s002.zip › Supplemental Figures S2/Figure13.png]

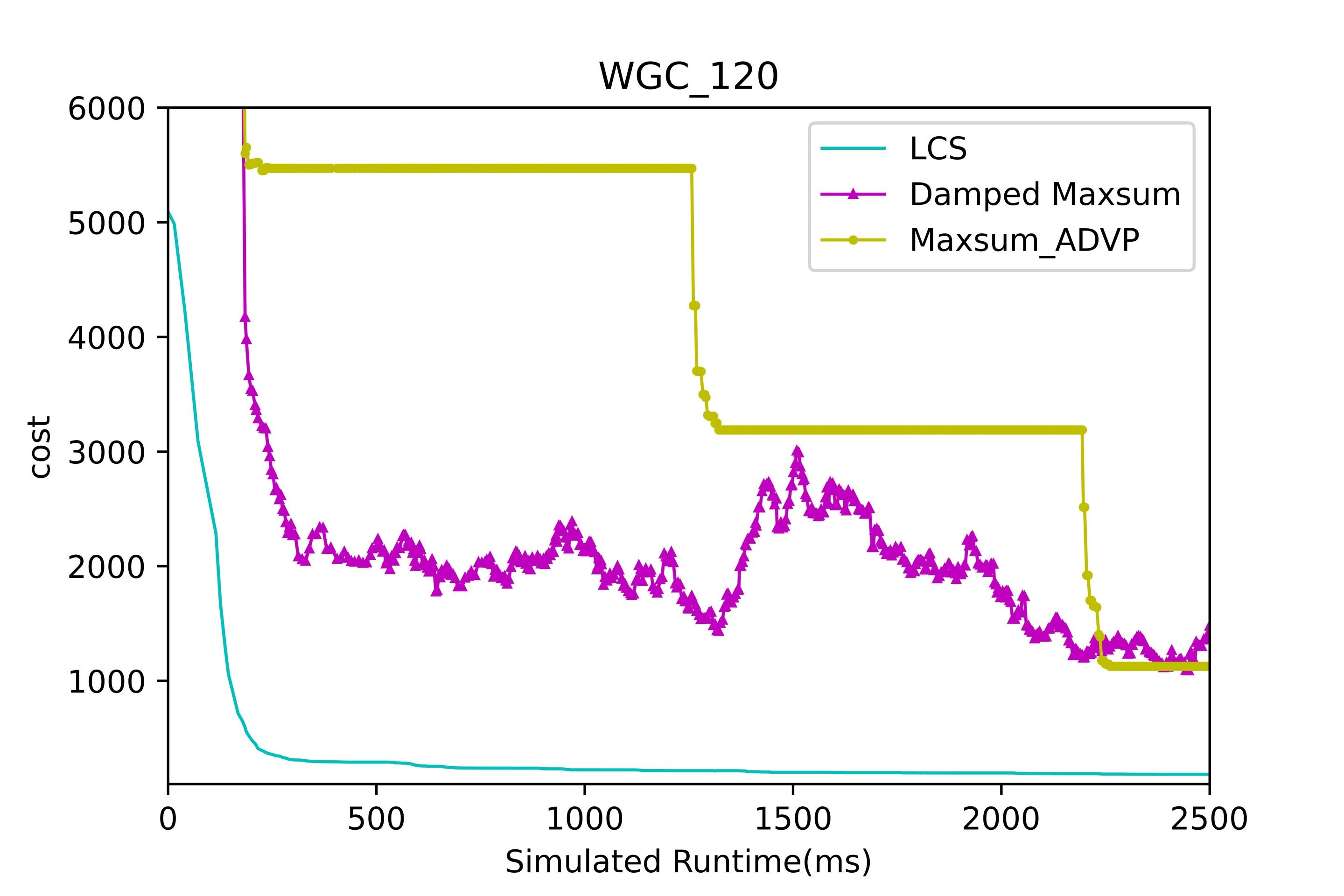

Supplement: Supplemental Information 2 [file peerj-cs-09-1296-s002.zip › Supplemental Figures S2/Figure14.png]

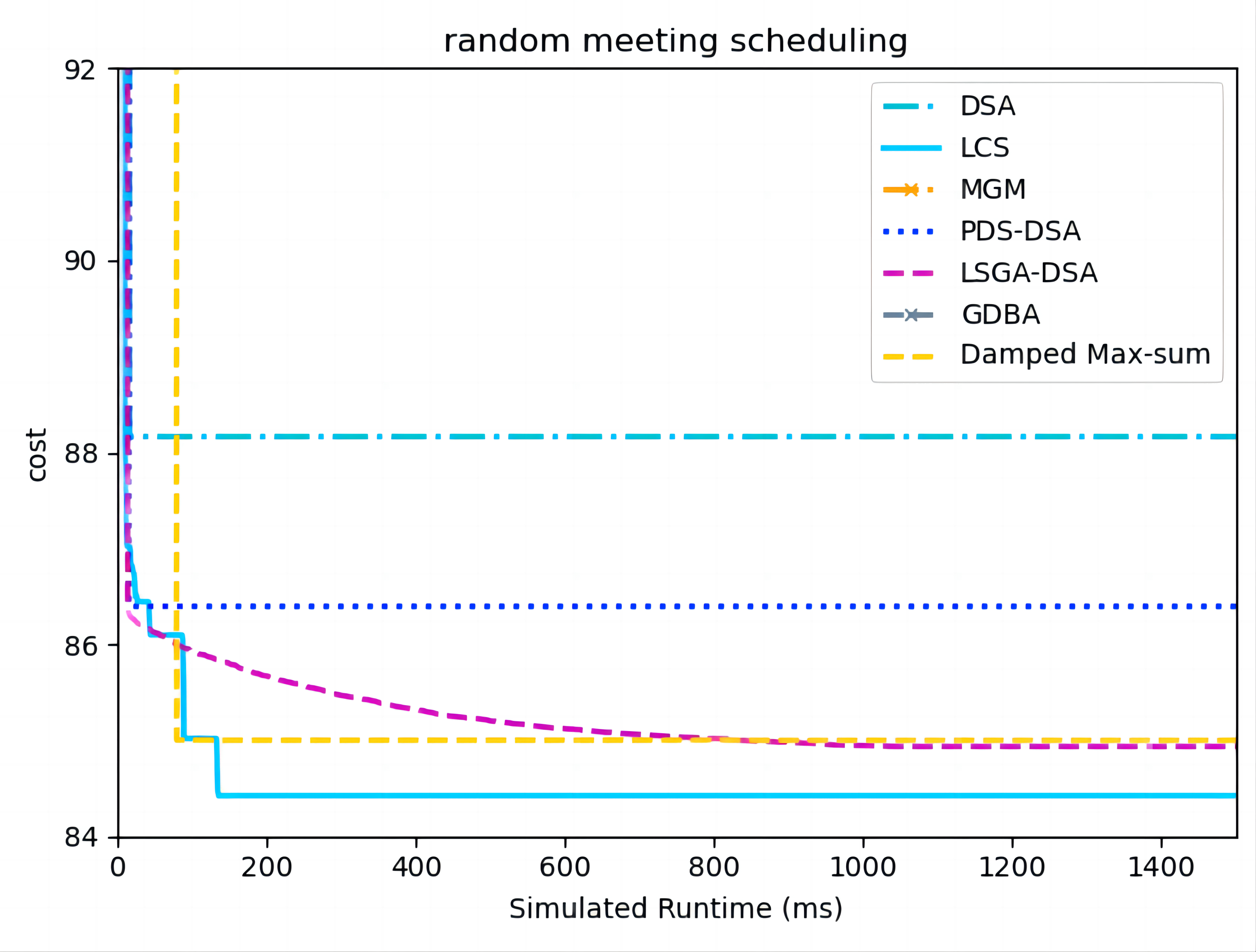

Supplement: Supplemental Information 2 [file peerj-cs-09-1296-s002.zip › Supplemental Figures S2/Figure15.png]

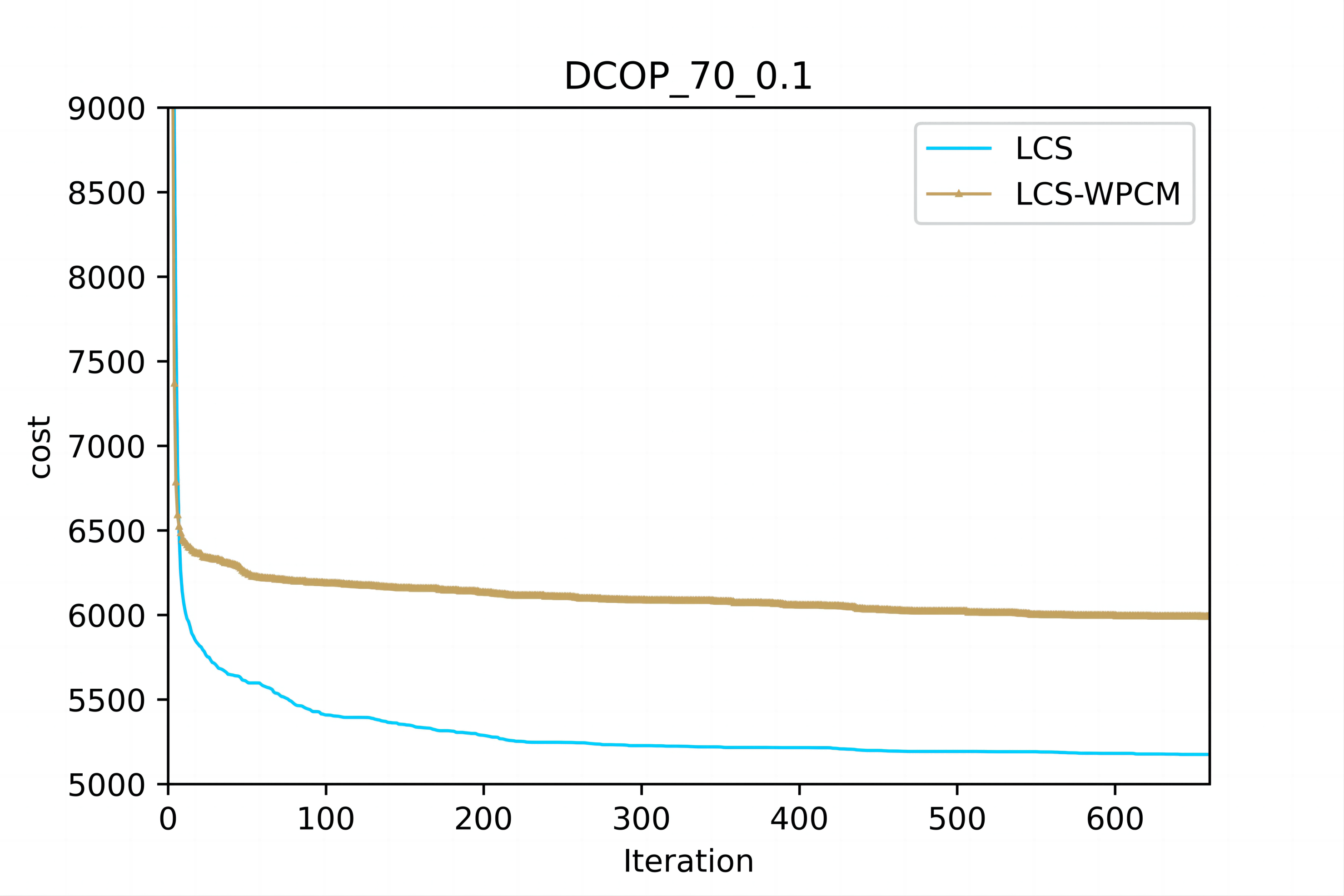

Supplement: Supplemental Information 2 [file peerj-cs-09-1296-s002.zip › Supplemental Figures S2/Figure2.png]

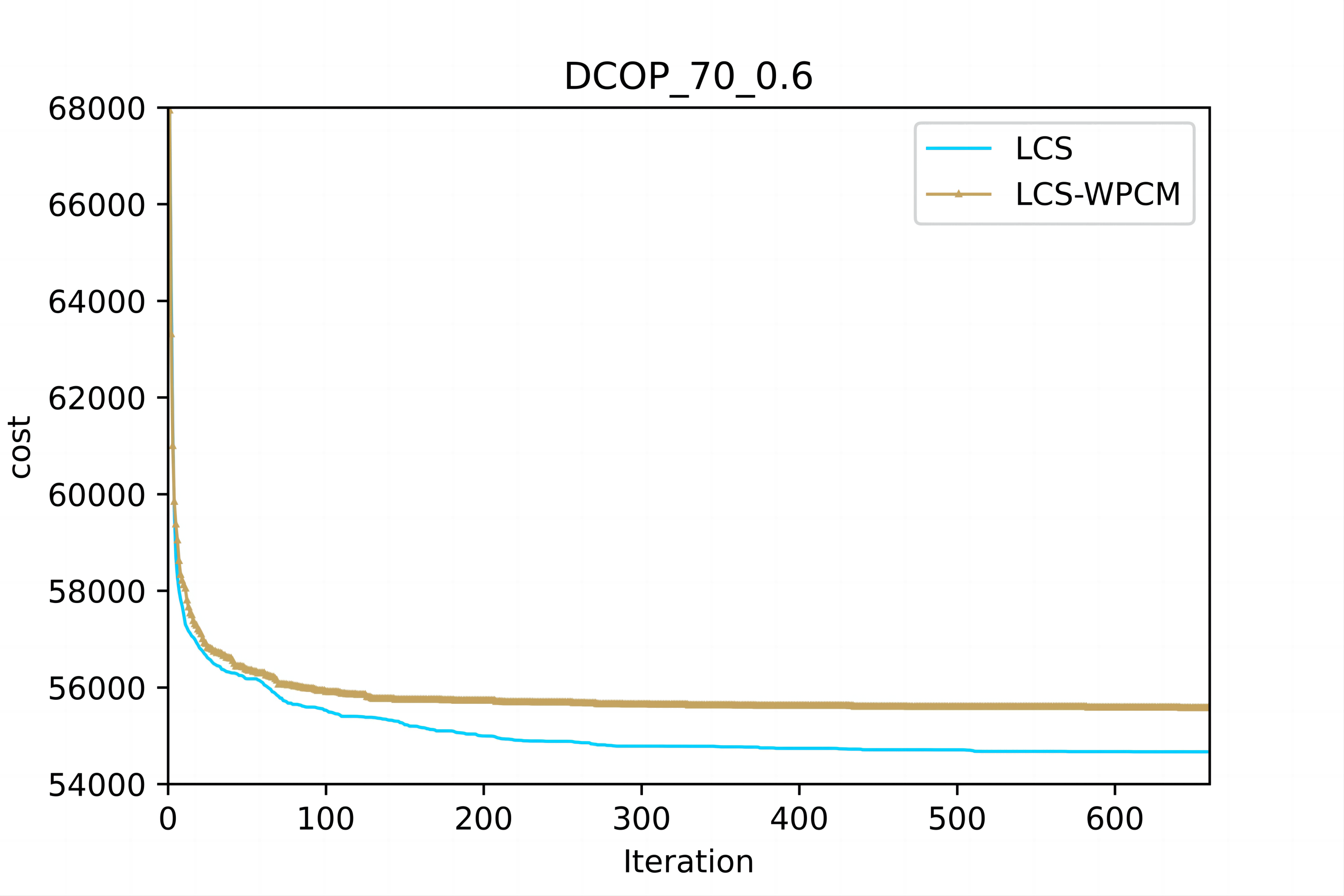

Supplement: Supplemental Information 2 [file peerj-cs-09-1296-s002.zip › Supplemental Figures S2/Figure3.png]

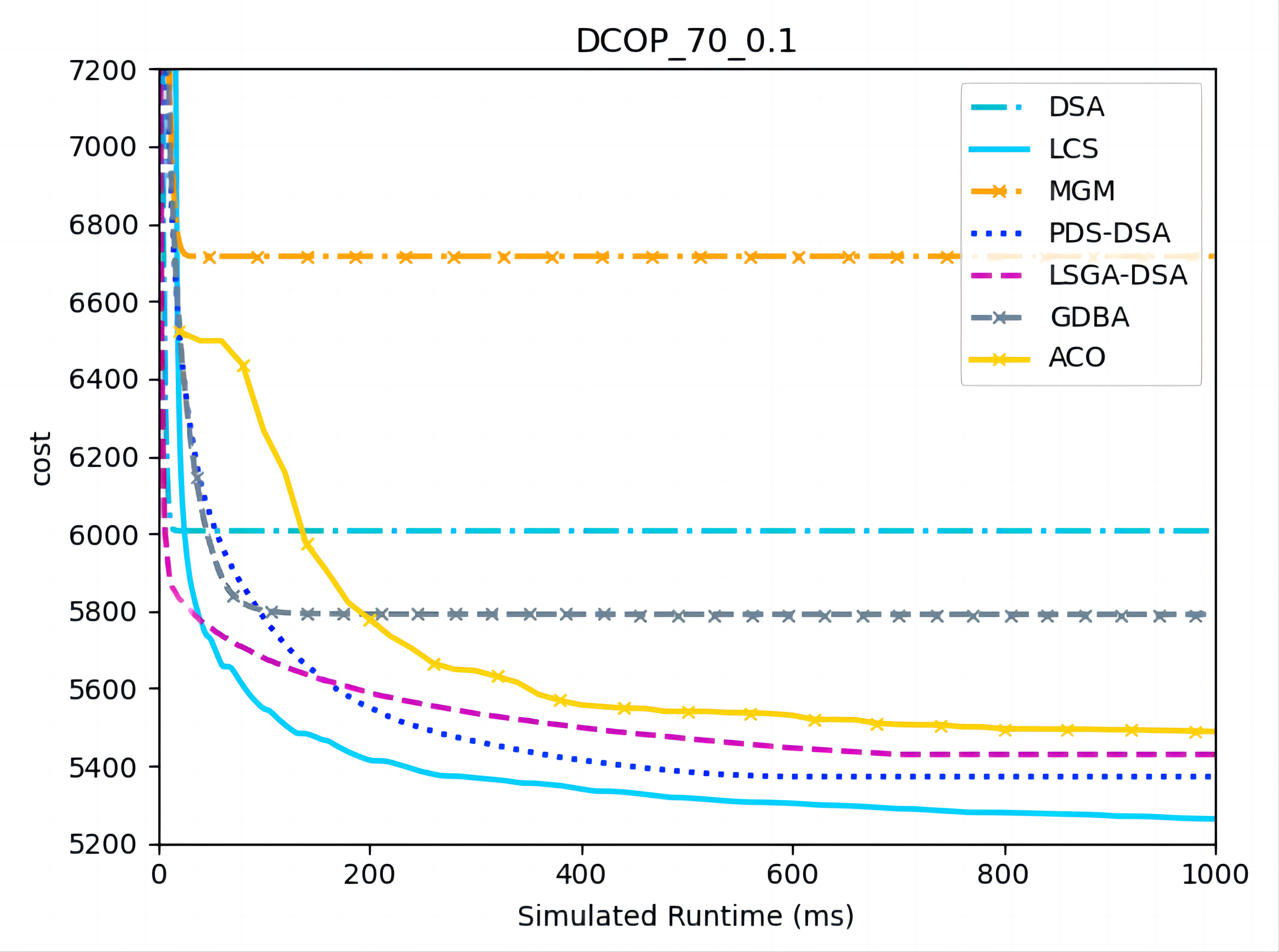

Supplement: Supplemental Information 2 [file peerj-cs-09-1296-s002.zip › Supplemental Figures S2/Figure4.png]

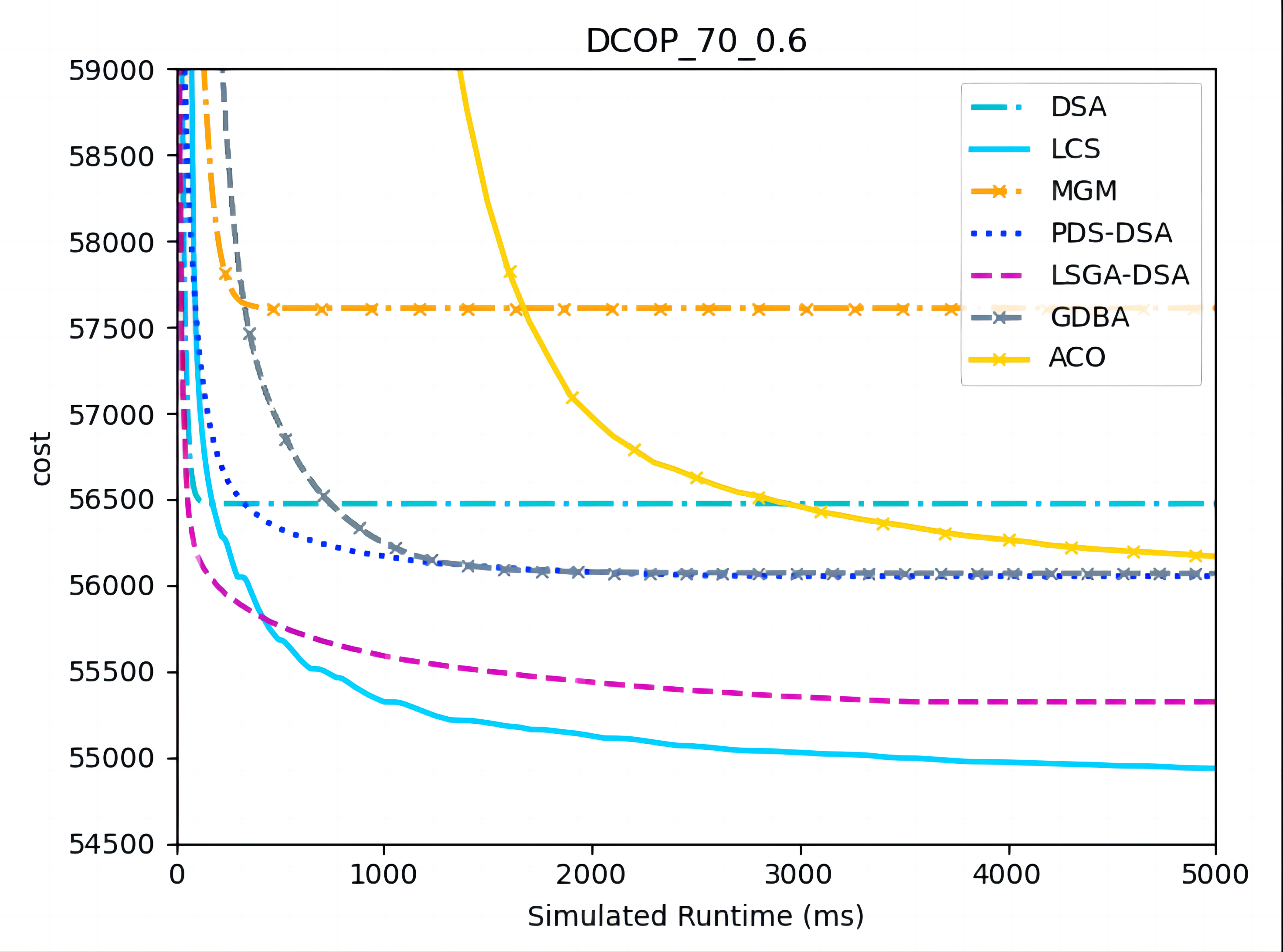

Supplement: Supplemental Information 2 [file peerj-cs-09-1296-s002.zip › Supplemental Figures S2/Figure5.png]

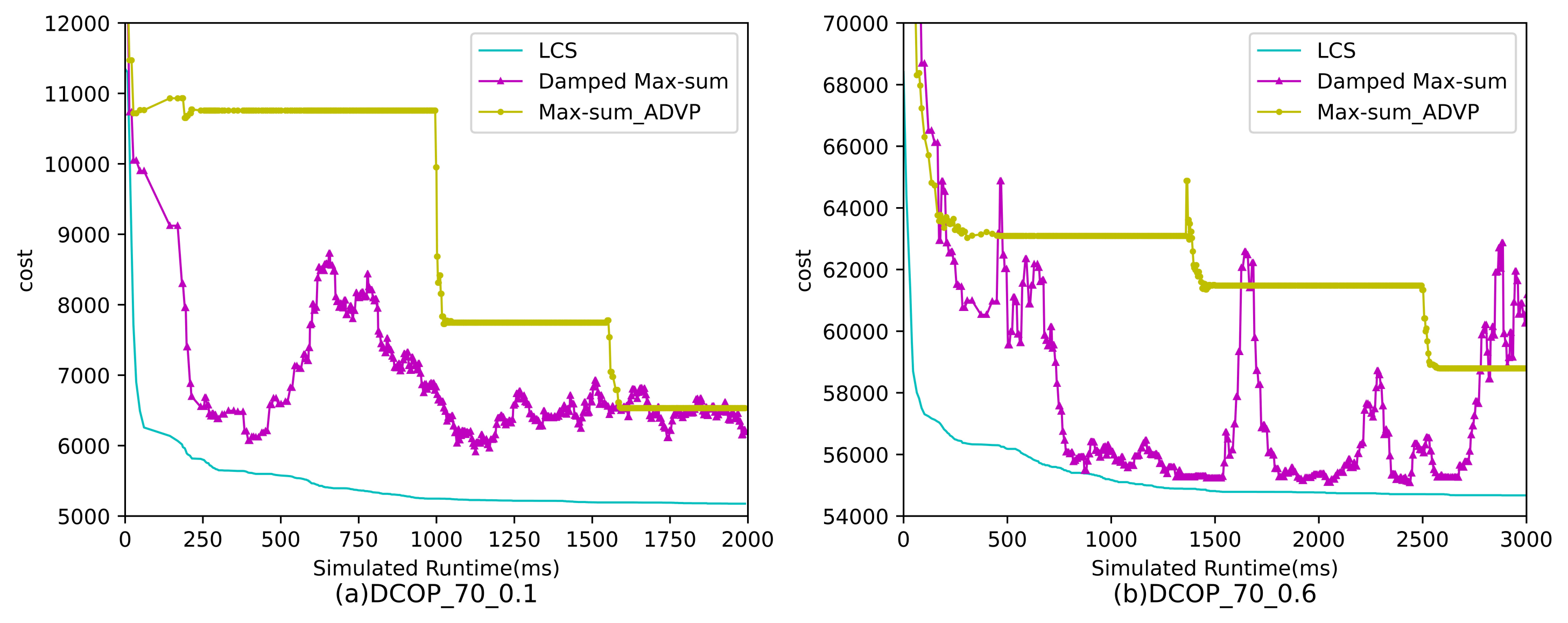

Supplement: Supplemental Information 2 [file peerj-cs-09-1296-s002.zip › Supplemental Figures S2/Figure6.png]

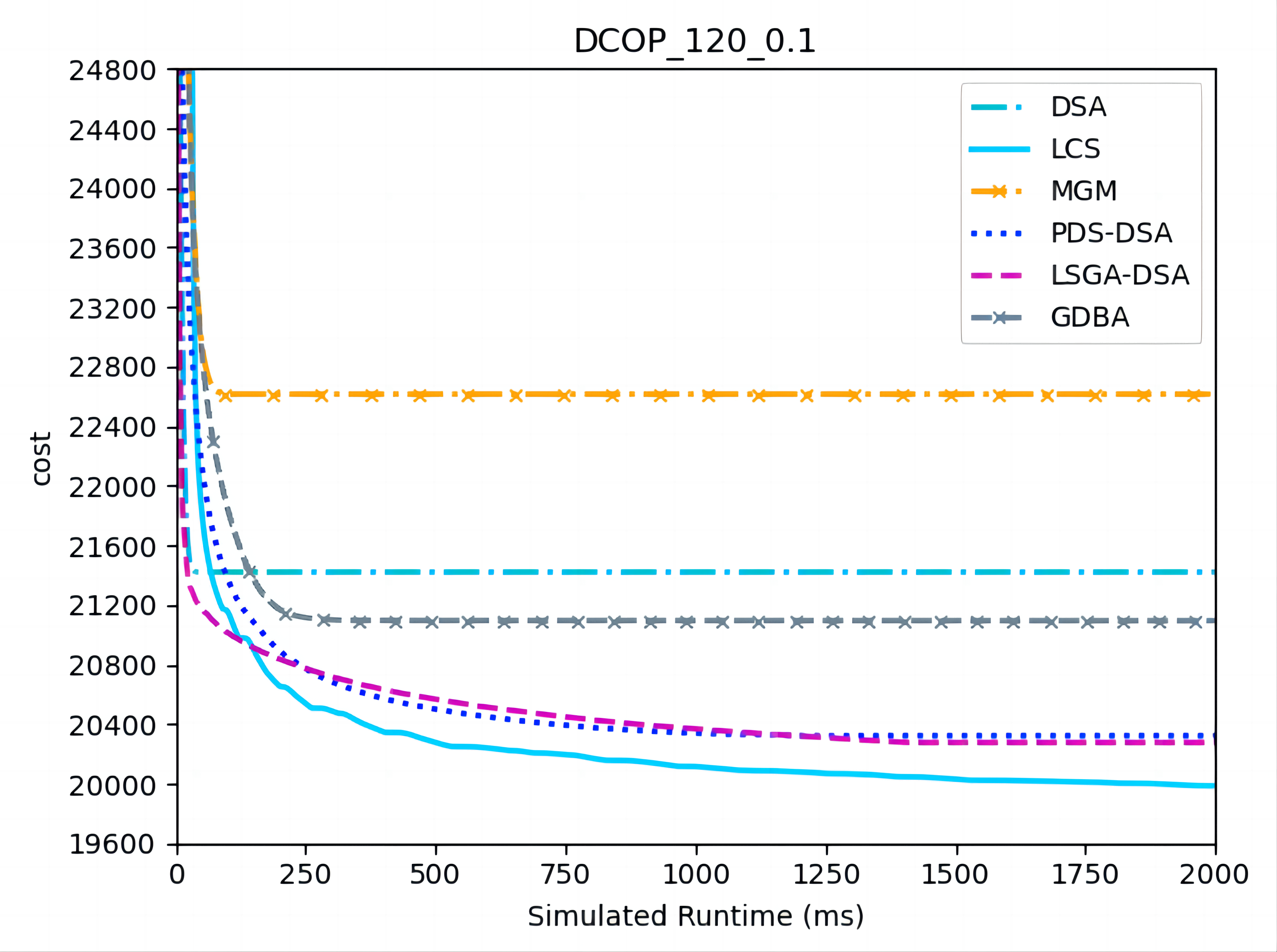

Supplement: Supplemental Information 2 [file peerj-cs-09-1296-s002.zip › Supplemental Figures S2/Figure7.png]

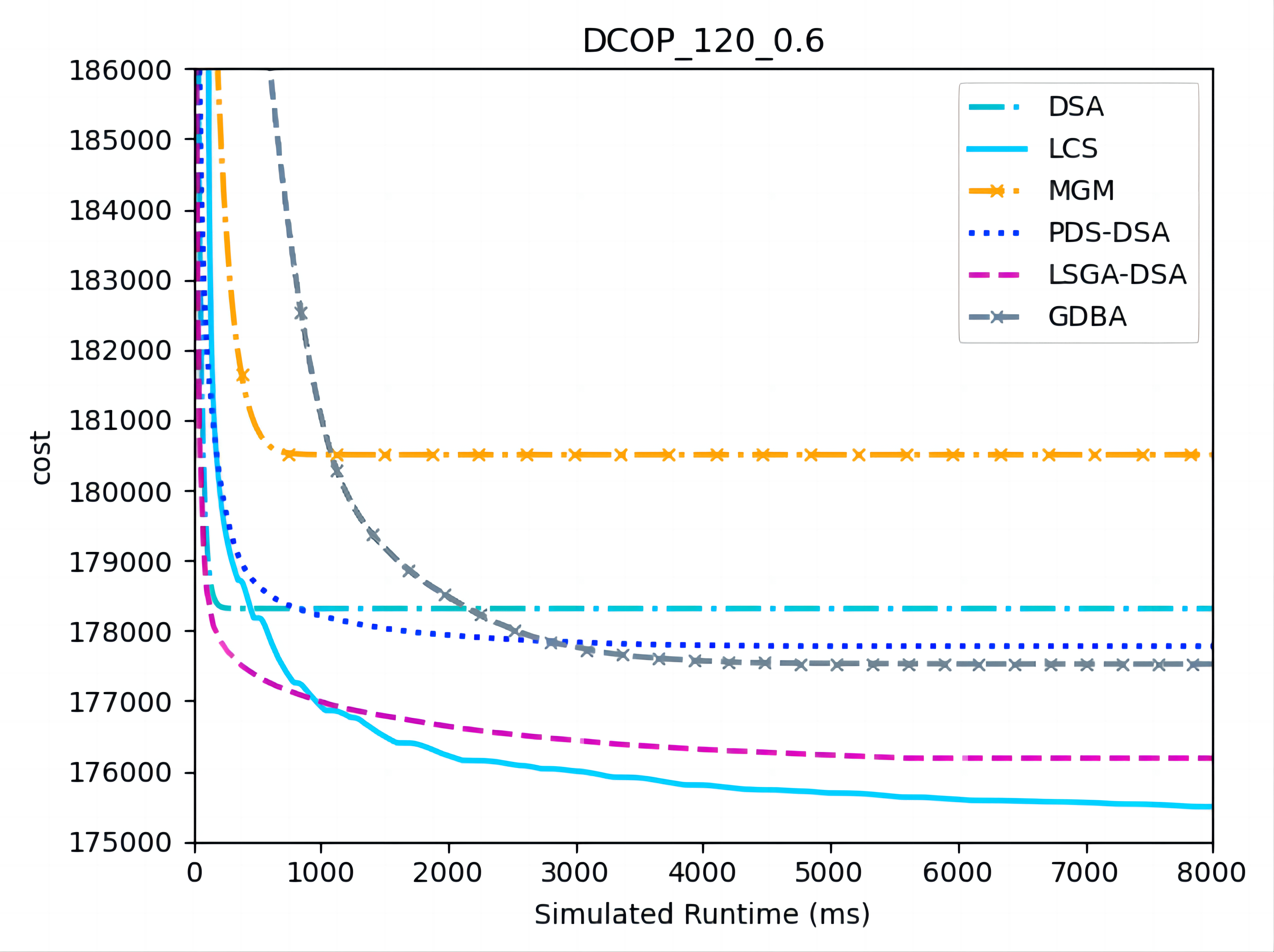

Supplement: Supplemental Information 2 [file peerj-cs-09-1296-s002.zip › Supplemental Figures S2/Figure8.png]

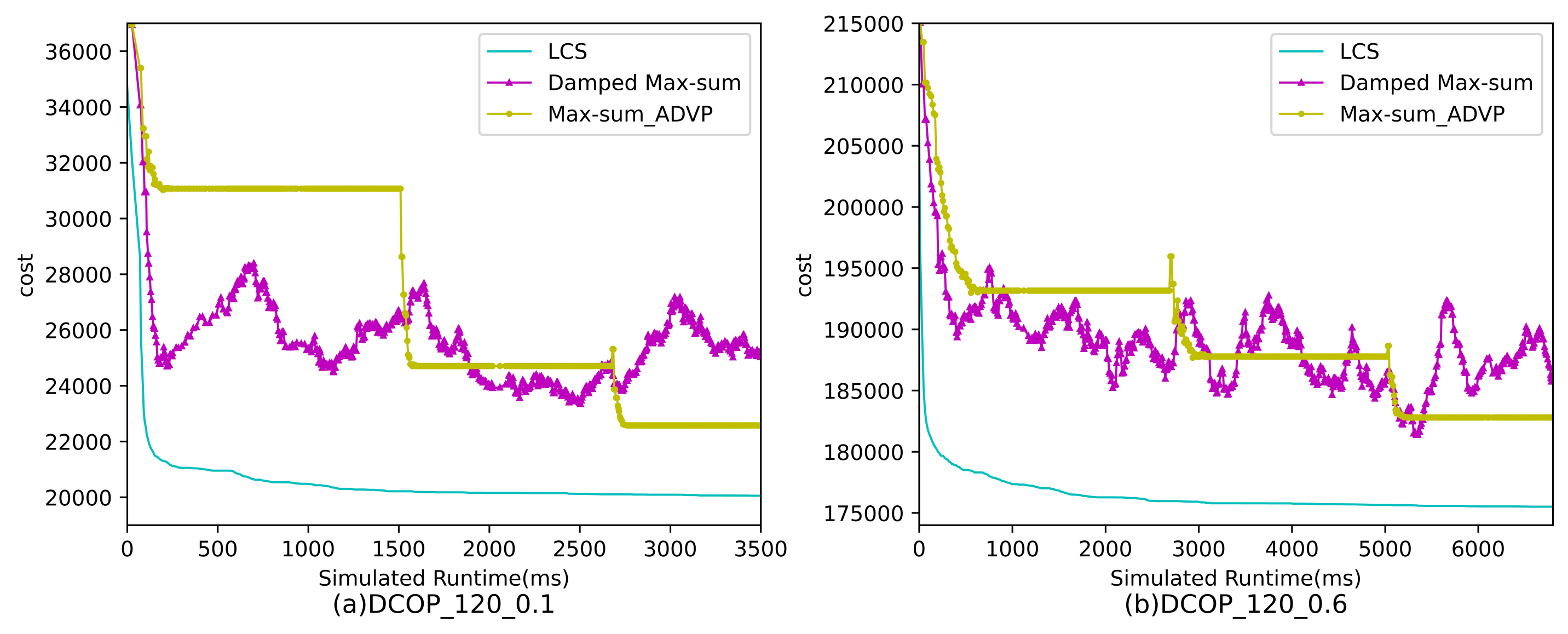

Supplement: Supplemental Information 2 [file peerj-cs-09-1296-s002.zip › Supplemental Figures S2/Figure9.png]
